# Multimodal phototherapy for Glioblastoma: from mechanistic action to synergistic delivery and therapeutic strategies

**DOI:** 10.1186/s12951-026-04441-y

**Published:** 2026-04-24

**Authors:** Gaurisha Alias Resha Ramnath Naik, Deepanjan Datta, Ritu Kudarha, Bhupendra Prajapati, Mital Patel, Varadharajan Srinivasan, Namdev Dhas

**Affiliations:** 1 Department of Pharmaceutics, Manipal College of Pharmaceutical Sciences, Manipal Academy of Higher Education, Manipal, India; 2 Department of Biotherapeutics Research, Manipal Academy of Higher Education, Manipal, India; 3 Department of Pharmaceutics, Parul Institute of Pharmacy, Faculty of Pharmacy, Parul University, Waghodia, Vadodara, Gujarat India; 4 Shobhaben Pratapbhai Patel School of Pharmacy and Technology Management, SVKM’s Narsee Monjee Institute of Management Studies (NMIMS) Deemed to be University, Mumbai, India; 5 Manipal Institute of Technology, Manipal Academy of Higher Education, Manipal, India

**Keywords:** Brain cancer therapy, Intranasal administration, Photothermal therapy, Photodynamic therapy, Targeted multimodal therapy

## Abstract

Glioblastoma (GBM) is the most aggressive primary malignant brain tumor, with a median overall survival of only 14–16 months despite maximal safe resection, radiotherapy, and temozolomide. The blood–brain barrier, infiltrative growth pattern, and tumor microenvironment-mediated therapeutic resistance severely limit the efficacy of conventional and emerging therapies. Phototherapy, including photodynamic therapy (PDT) and photothermal therapy (PTT), offers spatially selective tumor ablation through light activation, minimal invasiveness, and the ability to trigger immunogenic cell death. Recent advances in second- and third-generation photosensitizers, near-infrared-absorbing photothermal agents, and multifunctional nanoplatforms have substantially improved tumor-specific accumulation and therapeutic indices in preclinical GBM models. Intranasal administration has emerged as a clinically attractive, noninvasive route to bypass the blood–brain barrier and deliver photosensitizers and photothermal agents directly to the brain. Preclinical studies have demonstrated that combining PDT or PTT with temozolomide, immune checkpoint inhibitors, or ferroptosis inducers yields synergistic antitumor effects, prolongs survival and abscopal responses, and reduces postsurgical recurrence in orthotopic GBM models. Despite these encouraging outcomes, clinical translation is currently hindered by the depth of light penetration in the human brain, the nonuniform distribution of the drug inside the tumor, and the lack of systematic light delivery methods. To date, few phase one clinical trials of PDT have been conducted, and the findings provide the possibility of PDT intervention to increase survival rates to a certain extent. This review critically tabulates and reports the principles and approaches for the translation of multimodal phototherapy for the treatment of glioblastoma.

**Graphical Abstract:**

## Highlights

Phototherapy offers targeted, minimally invasive treatment for glioblastoma (GBM) with reduced systemic toxicity.

Intranasal delivery bypasses the blood-brain barrier (BBB) but requires improved tumor targeting and synchronized light activation.

Recent advancements in nanotechnology and smart photosensitizers have improved the selectivity and efficacy of PDT and PTT.

Key challenges for GBM phototherapy include light penetration and drug retention.

The future of GBM treatment may rely on combining phototherapy with personalized methods and collaborative research.

## Graphical abstract

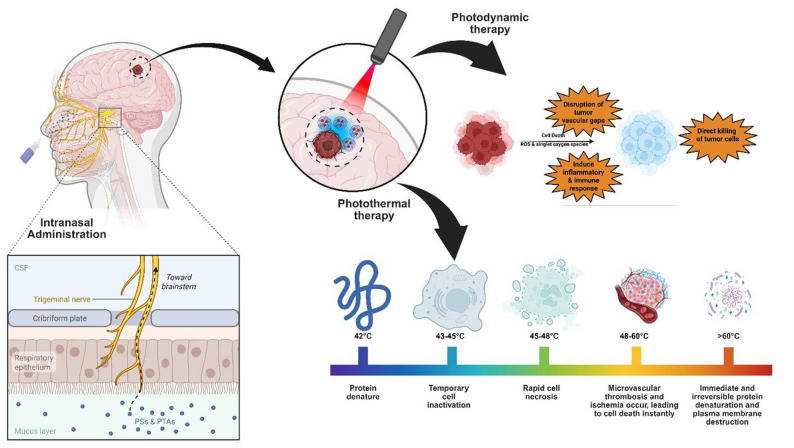

## Introduction

Glioblastoma multiforme (GBM) is known as the most common type of primary malignant brain tumor of the central nervous system (CNS) [1]. GBM is a highly aggressive and malignant brain tumor that is often located in the cerebral hemisphere [2]. Despite its rarity, with a worldwide incidence of no more than 10 cases per 100,000 people, GBM still accounts for approximately 16% of primary brain tumors and 54% of all gliomas. Its survival rate is barely 14–15 months post-diagnosis due to its poor prognosis standards and is therefore a serious health concern [3, 4]. It occurs mainly in individuals aged 40–60 years and predominantly affects males rather than females [5, 6].

The conventional treatment of GBM includes maximal surgical removal of the tumor followed by temozolomide (TMZ) chemotherapy and radiation therapy [7, 8]. The challenges in GBM therapy include incomplete blood‒brain barrier (BBB) permeability, apoptosis resistance, early drug release, nonspecific tumor targeting, and secondary toxicity in normal cells. The advanced vasculature, invasive properties, and complex genetic, molecular, and cellular processes underlying GBM are responsible for its high recurrence rate and drug resistance [9]. Efforts are underway to overcome such hindrances through new methods in medicine. Intranasal/intracerebral administration of drugs, loading drugs into nanocarriers, and targeting ligands attached to such nanocarriers are some of the recent BBB-avoiding approaches for GBM therapy [10]. New therapies are also being developed via combination treatments, such as photothermal, photodynamic, or sonodynamic therapy, together with chemotherapy.

Phototherapy includes photodynamic therapy (PDT), which generates toxic reactive oxygen species, and photothermal therapy (PTT), which produces lethal hyperthermia to ablate tumors [11]. These light-activated approaches overcome blood-brain barrier and therapeutic resistance when combined with nanotechnology [12]. Phototherapy alone has limited efficacy and therefore has to be used in conjunction with conventional therapies. It has attracted considerable interest and has been investigated in recent years because of its highly effective nature in clinical treatment, its minimal invasiveness and minimal side effects [13]. The development of light-mediated approaches using nanoparticles and photosensitizers (PSs) as diagnostic and therapeutic agents is crucial for successful cancer therapy. Owing to its novel mechanism of action, it does not cause cross-resistance with other therapies. Hence, combination therapies can be used to improve treatment efficiency while minimizing adverse effects and systemic toxicity [14].

Nevertheless, phototherapy also has many limitations, including the inability to selectively target malignant cells due to the existence of endogenous chromophores in normal tissues and extremely high levels of irradiance [15, 16]. These limitations might be mitigated by focusing the light solely in the target tissue, thus making tumors selective. In addition, PSs are used in phototherapy to enhance the therapeutic effects of light exposure by stimulating their preferential accumulation in target tissues and photon absorption [17]. Moreover, combining phototherapy with other interventions might also be viewed as a specific modality for treating GBM, a strategy that addresses treatment-related resistance and increases the level of therapeutic efficacy and clinical outcomes [18]. This approach can be effectively combined with traditional chemotherapy, thus allowing the sensitization of tumor cells to chemotherapeutic drugs while allowing dose cuts to occur without negatively affecting therapeutic outcomes [19]. The literature has demonstrated that PDT in combination with TMZ leads to better apoptosis in GBM than either of the other two treatments alone [20]. In addition, the nanoparticles used in phototherapy can be used to entrap drugs so that they can be specifically delivered to the tumor site. Moreover, the combination of radiation therapy with phototherapy has been demonstrated to have a significant advantage over monotherapy [21]. Phototherapy enhances the sensitivity of GBM cells to radiation by increasing cellular oxidative stress and damaging their cellular components, increasing their susceptibility to radiation-induced cytotoxicity [22].

Research is being carried out to identify potential drug combinations involving phototherapy and the repurposing of already developed drugs, in addition to conventional chemotherapy, to achieve synergistic effects and inhibit GBM cell viability [18]. Therefore, the synergistic effects of targeted phototherapy and other therapeutic approaches can efficiently address the drawbacks of GBM therapy. By using the distinct potential of all therapies, the cytotoxicity of an approach can be minimized, while its efficacy can be maximized via modern research in nanotechnology and immunotherapy [23].

**Fig. 1:**
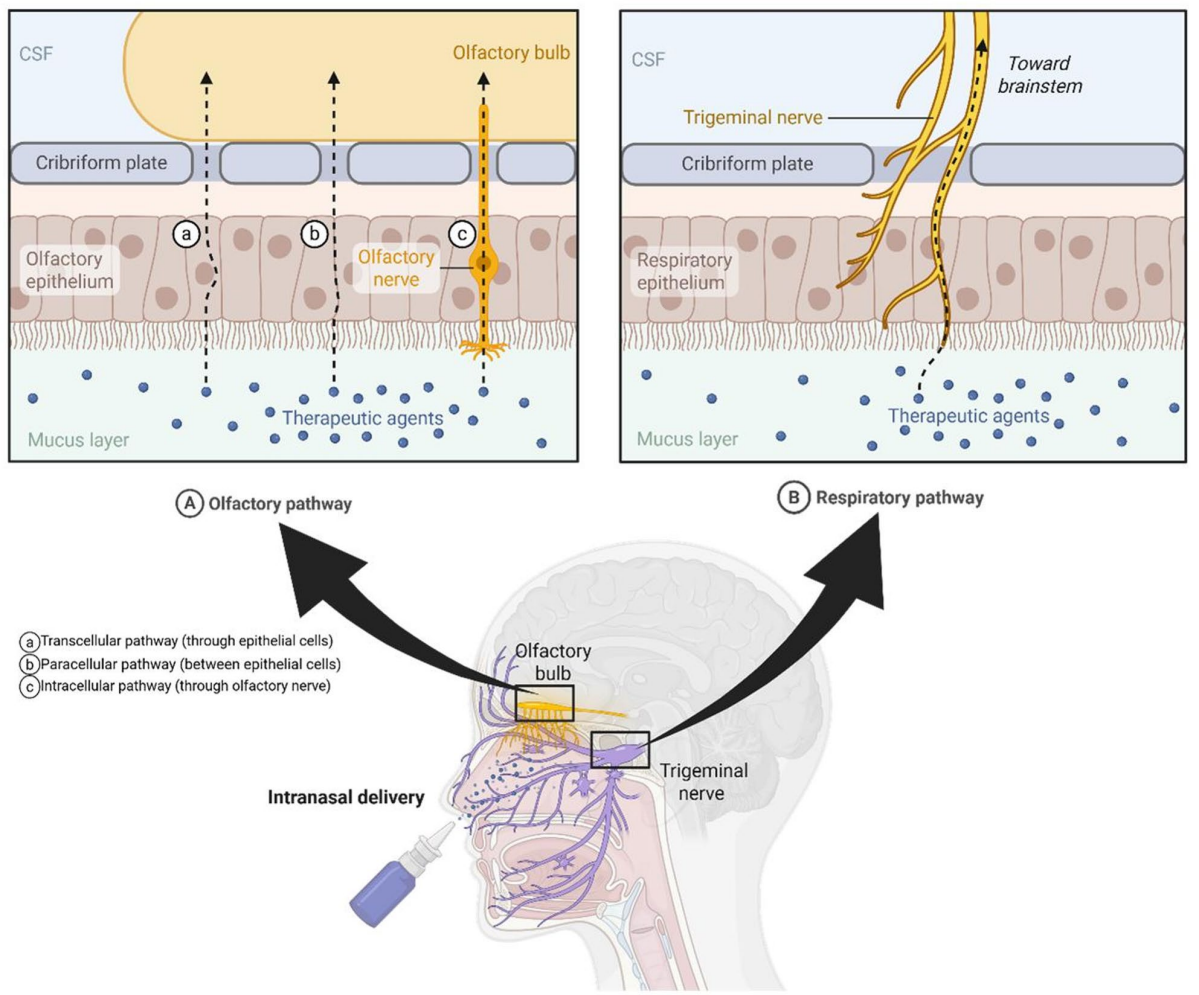
Schematic illustration of intranasal drug delivery pathways to the brain. Drugs administered into the nasal cavity can reach the central nervous system via the olfactory nerve pathway (nose-to-brain) and the trigeminal nerve pathway, thereby bypassing the BBB and achieving direct nose‒to-brain transport. Created in BioRender. Dhas, N. (2025). https://BioRender.com/dwry7gc

Conversely, the effectiveness of these multifaceted therapies depends on their ability to overcome drawbacks such as BBB permeability and tumor heterogeneity. A novel strategy for overcoming BBB limitations is intranasal delivery, which allows direct, efficient transfer of therapeutic agents into the brain [24]. Furthermore, this drug delivery route also differs in that it improves drug bioavailability while reducing side effects, leading to the development of yet another new strategy [25]. Intranasal drug delivery uses the olfactory and trigeminal nerves, which translocate drugs administered through the nasal route toward the CNS with reduced systemic drug circulation as well as first-pass hepatic drug delivery, as illustrated in Fig. 1.

Studies have demonstrated that when interventions are administered intranasally, they accumulate effectively in tumor cells and significantly prolong median survival in treated rats compared with controls. The intranasal delivery system provides direct entry into cerebral tissues and reduces neurotoxicity to nearby healthy cells [26]. The advantages of this route of administration are not limited to bypass of the BBB. Compared with intravenous delivery, it is convenient and self-administered, thus increasing patient compliance [27]. Additionally, intranasal administration has been demonstrated to increase the bioavailability of other chemotherapeutic agents and ultimately lead to tumor regression in preclinical models. The efficacy and therapeutic outcome are further improved through the incorporation of nanocarriers [28]. Overall, intranasal delivery offers a rapid approach for effective drug delivery to brain tumors by bypassing all traditional barriers and facilitating the rapid delivery of therapeutics to affected areas [29]. More research and clinical studies are necessary to elucidate the potential of phototherapy as a treatment for brain cancer [30]. Overcoming these issues is crucial for making this modality a viable treatment choice for patients with GBM.

The effectiveness of PDT and PTT depends on the regulated contact between electromagnetic radiation and biological tissues. Light propagation is altered by reflection scattering, absorption and transmission when light penetrates tissue [31, 32]. X-rays have high frequency and intensity, enabling easy penetration through the body and revealing structures, unlike ultraviolet radiation, which is highly absorbed by water and therefore reduces its penetration depth [33]. Near-infrared (NIR) light, which has comparatively low values of absorption and scattering coefficients between 600 and 1300 nm, offers greater penetration in soft tissues [34].

This review summarizes phototherapy and its use in GBM treatment, and most of the discussion centers on its mechanisms, procedures, and application as a multimodal therapy-directed therapy. It addresses photodynamic and photothermal therapy, its action on cancer cells, and outcomes. The role of combined therapeutic methods, the benefits of intranasal drug delivery to bypass the BBB, and future innovations in phototherapeutic methods are also discussed. Finally, further research that could help optimize phototherapy and improve the clinical outcome of GBM patients is needed.

## Literature search methodology

### Search strategy

This narrative review was conducted following a systematic literature search strategy aligned with PRISMA recommendations for study identification, screening, and selection. A comprehensive search was performed across four major electronic databases: PubMed, Scopus, Web of Science, and Google Scholar. The search covered peer-reviewed original research articles, review papers, and clinical trial reports published between 2000 and March 2026. The search strategy combined Medical Subject Headings (MeSH) terms (where applicable) with free-text keywords using appropriate Boolean operators. The primary search string was: (glioblastoma OR GBM OR high-grade glioma) AND (phototherapy OR photodynamic therapy OR PDT OR photothermal therapy OR PTT) AND (nanoparticles OR nanotechnology OR photosensitizer OR intranasal delivery OR nose-to-brain OR multimodal).

Additional targeted searches included terms such as “phototherapy clinical trials glioblastoma”, “nanoparticle-mediated photodynamic therapy brain tumor”, “5-ALA PDT glioblastoma”, and related combinations. The aim of the search was to identify all relevant studies involving PDT, PTT, or multimodal phototherapy in GBM or high-grade glioma, with emphasis on nanotechnology-based delivery systems, photosensitizers, intranasal routes, and synergistic approaches. Only original research articles, reviews, and clinical trial reports published in peer-reviewed journals and written in English were included.

### Inclusion and exclusion criteria

#### Inclusion criteria

Studies were included if they met all the following criteria:

- Focused on PDT, PTT, or multimodal phototherapy in the context of GBM or high-grade glioma (in vitro, in vivo, or clinical settings).
- Involved nanotechnology-based delivery systems, photosensitizers, photothermal agents, intranasal/nose-to-brain delivery, or synergistic combinations with other therapeutic modalities.
- Provided mechanistic insights, efficacy data, therapeutic outcomes, or translational perspectives relevant to GBM treatment.
- Were published in English with full text available and contained sufficient methodological or outcome details.

#### Exclusion Criteria

Studies were excluded based on any of the following criteria:

- Articles unrelated to light-mediated therapies or GBM/high-grade glioma.
- Conference abstracts, editorials, letters, book chapters, or non-peer-reviewed publications.
- Studies lacking sufficient methodological or outcome details on photodynamic/photothermal mechanisms, nanotechnology, intranasal delivery, or multimodal strategies.
- Duplicate publications, with preference given to the most complete or recent version.

### Study selection

All records retrieved from the database searches were exported and managed using reference management software. Duplicates were detected and removed. Following duplicate removal, titles and abstracts were independently screened. Potentially eligible records underwent full-text evaluation. Reference lists of included articles and relevant reviews were manually examined to identify additional eligible studies. Priority was given to publications from the past five years (2020–2025) to reflect recent advances in multimodal nanoplatforms, intranasal delivery, and early clinical translation.

**Fig. 2:**
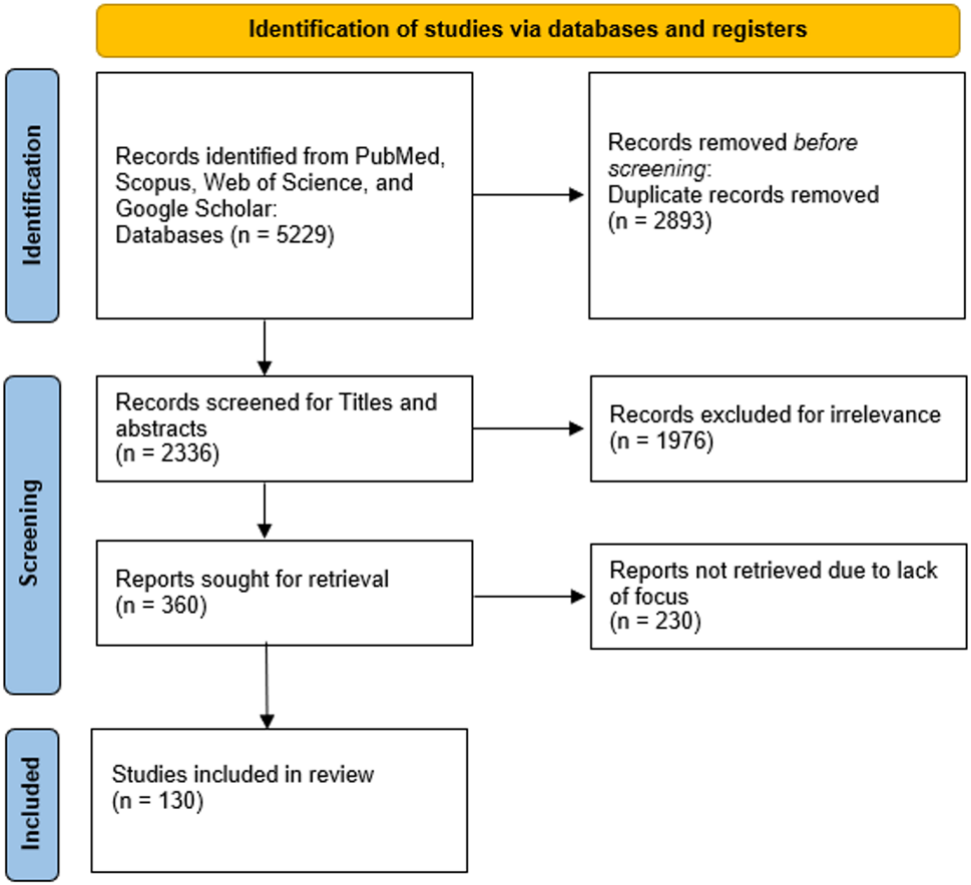
PRISMA flow diagram illustrating the identification, screening, eligibility assessment, and final inclusion of studies from database searches and additional manual reference screening for this article

A PRISMA-style flow diagram depicted in Fig. 2 was constructed to summarize the study identification and selection process. The database searches initially identified 5,229 records. After duplicate removal (2,893 records), 2,336 unique records underwent title and abstract screening, of which 1,976 were excluded for irrelevance or failure to meet inclusion criteria. Full-text assessment was performed on the remaining 360 articles, leading to the exclusion of 230 records due to a lack of focus on GBM phototherapy, non-English language, or absence of relevant data on photodynamic/photothermal mechanisms, nanotechnology, intranasal delivery, or insufficient methodological details. Ultimately, 130 studies were included in the qualitative synthesis. The diagram further incorporates records identified through manual reference screening and targeted searches for clinical trials and patents.

### Data synthesis

Due to the substantial heterogeneity among the included studies, with respect to the types of photosensitizers and photothermal agents, delivery routes, light parameters (wavelength, fluence, irradiance), tumor models (cell lines vs. orthotopic vs. subcutaneous), and reported outcome measures, as well as the inclusion of preclinical, clinical, and review articles, a quantitative meta-analysis was not feasible. Therefore, a qualitative narrative synthesis was performed using a thematic approach. The extracted data were organized into key themes covering the mechanistic principles of phototherapy, nanotechnology-enabled delivery strategies (including intranasal administration), preclinical and early clinical efficacy findings, synergistic multimodal combinations, and major barriers to clinical translation. This thematic framework enabled a comprehensive, critical overview of the current state and future prospects of multimodal phototherapy for glioblastoma.

## Core mechanisms of phototherapy

Phototherapy has gained increasing importance as a therapeutic option for treating brain malignancies, especially with the use of PDT and PTT [19]. The modality uses light, photothermal agents (PTAs), and PSs to destroy malignant cells and thus offers a noninvasive alternative to existing methods such as surgery and chemotherapy.

In PDT, PSs accumulate preferentially in tumor cells and are excited via photons at wavelengths of 630–800 nm, that is, the red or near-NIR light spectrum [16, 35], which then transfer energy to ground-state oxygen and generate highly reactive singlet oxygen and other ROS [36]. The activation of PSs results in the production of reactive oxygen species (ROS), which are powerful initiators of cellular injury that trigger tumor cell apoptosis or necrosis. The mechanism of excitation is based on the release of energy in the form of fluorescence and the transition to the excited triplet form, where the ROS, including singlet oxygen, are formed [37, 38]. The resulting oxidative stress causes permanent damage to lipids, proteins, and DNA, leading to permeabilization of the mitochondrial outer membrane, the release of cytochrome c and the initiation of caspase-dependent apoptosis [39]. Concurrently, ROS‑mediated injury to the tumor endothelium induces vascular thrombosis and ischemia, which amplifies cell death through secondary necrosis [36, 40]. Crucially, dying cells expose or release damage‑associated molecular patterns (DAMPs), such as calreticulin, ATP, and HMGB1, thereby converting immunologically “cold” glioblastoma into an inflamed microenvironment capable of priming antitumor CD8⁺ T‑cell responses [41] There is also some evidence that PDT can promote antitumor immune responses, which further adds to its therapeutic efficacy [42].

PTAs for PTT, such as metal nanoparticles or functionalized graphene oxide, use NIR light in the 700–1100 nm range for deep tissue penetration and accumulate in the tumor microenvironment (TME) via nonradiative relaxation [43, 44]. It initiates with the absorption of NIR photons by PTAs, which trigger a transition from the ground to the excited state, converting optical energy into thermal energy and producing localized hyperthermia, which eventually triggers cell apoptosis [45]. PTAs trigger endogenous apoptosis via protein denaturation and endoplasmic reticulum stress at 42–47 °C, whereas at 50 °C and above, immediate cell membrane rupture and coagulation cause exogenous necrosis [16]. Malignant cells are more vulnerable than normal cells due to disorganized vasculature and changes in metabolic needs [46]. Hyperthermia also promotes the release of heat shock proteins (HSP70/HSP90), potent danger signals, enhances the cross-presentation of tumor antigens by dendritic cells [40], and causes vascular thrombosis, ischemia, and protein denaturation in cancer cells [47]. Additionally, heat disrupts tumor vasculature and degrades the dense extracellular matrix formed by cancer-associated fibroblasts, further destabilizing the immunosuppressive glioblastoma microenvironment [48]. In addition, the conjugation of targeting ligands increases selectivity and affinity for tumor-specific biomarkers, thereby reducing off-target effects and limiting collateral damage to healthy tissues.

When PDT and PTT are combined or applied in sequence, mild hyperthermia from PTT increases membrane permeability and enhances tumor oxygenation, which significantly increases ROS generation during subsequent treatments [49]. The synergistic combination of PDT and PTT enhances therapeutic outcomes. Coadministration of PSs and PTAs creates a hyperthermic environment in the latter, which increases the permeability of tumor cell membranes and thus improves the uptake of chemotherapeutic agents and their overall effectiveness [50]. This combination significantly amplifies the immunogenic cell death and systemic antitumor immunity effects consistently observed in orthotopic GBM models [51]. Furthermore, localized hyperthermia enhances apoptosis and may contribute to the liberation of tumor antigens, therefore inducing a strong immunogenic reaction against GBM cells.

## Photodynamic Therapy (PDT) in Glioblastoma

**Fig. 3:**
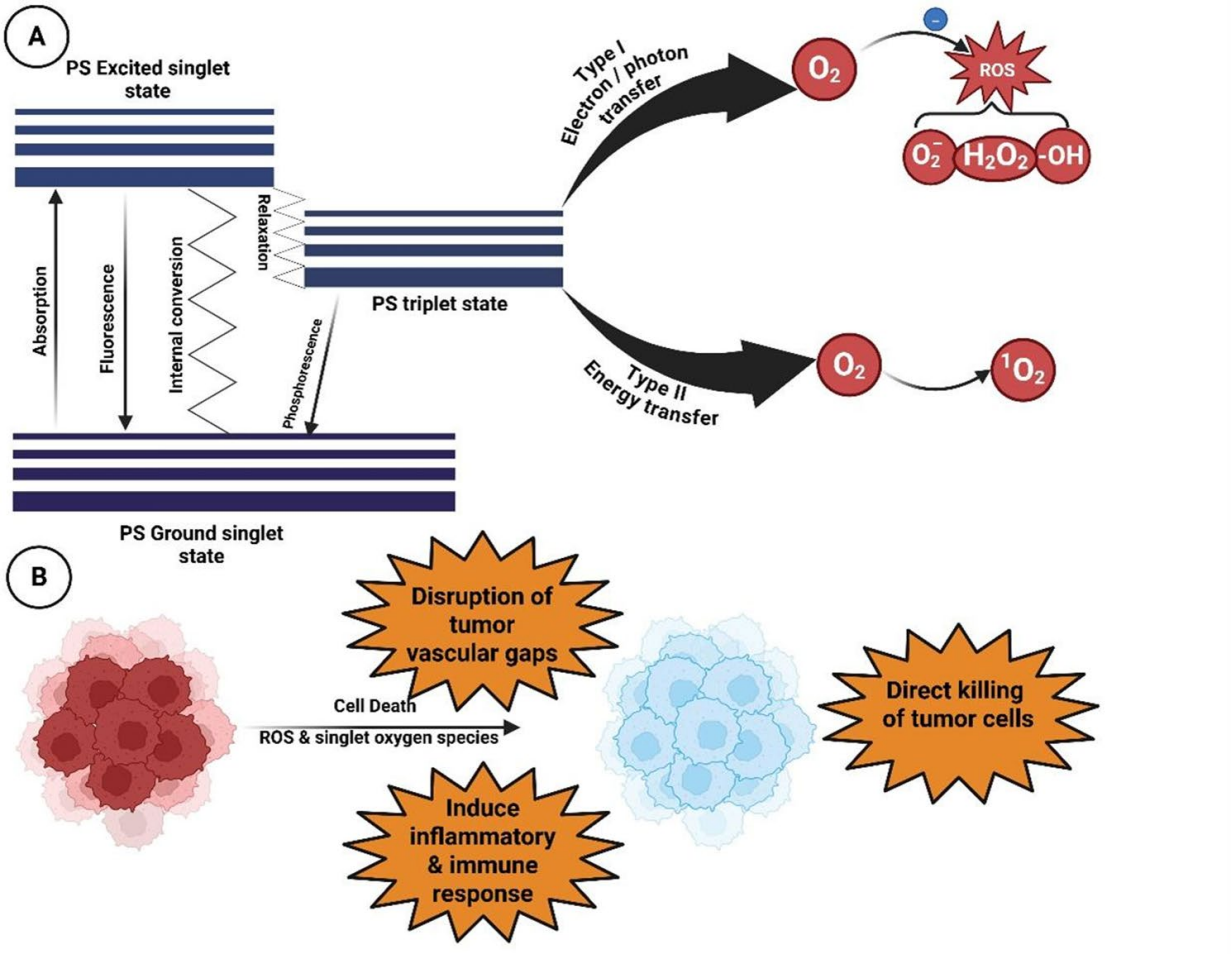
Mechanism of PDT. **(A**) A light-excited photosensitizer transfers energy to O₂, generating cytotoxic singlet oxygen and ROS. (**B**) ROS induce glioblastoma apoptosis/necrosis, vascular damage, and immunogenic cell death, accompanied by DAMP release. Created in BioRender. Dhas, N. (2025). https://BioRender.com/zqjv3r5

The term PDT was first used by medical student Oscar Raab, who worked with Professor Hermann von Tappeiner over a century ago [52]. Originally, PDT was developed to treat dermatological diseases wherein skin lesions are exposed to light and are easily assessed visually. Currently, PDT aids in the treatment of various diseases. The main principle of PDT is based on the localization of PSs within the TME, as discussed in Sect. "Core Mechanisms of Phototherapy" and depicted in Fig. 3 [35, 53].

### Types of photosensitizers

A good PS is characterized by high selectivity and high cytotoxicity toward the targeted tissue in the presence of light. While research is constantly developing more organ-specific PSs, they must undergo a series of clinical trials [54]. The damage caused by PDT is dependent on the type and concentration of PS used and the wavelength of the light irradiation. Although superficial lesions on the mucous membrane and skin have been effectively treated by PDT clinically, lesions located deeper into the tissue pose a challenge because of the difficulty in accessing them and the limited penetration depth of light [38]. Studies have also been conducted on modifying available PSs to minimize their potential side effects on healthy tissues and to enable the delivery of these PSs to targeted cells. Various PSs are being tested and employed in clinical trials for GBM therapy [55]. PS is characterized by toxicity, time, and effectiveness in terms of its concentration in the tissue, range of light absorption, and depth of tissue penetration. Currently, no PS is available that addresses all these aspects, especially in the case of brain tumors. PSs can be categorized into three generations. To provide a clear summary of the classification of photosensitizers and their key properties relevant to phototherapy, the following table, Table 1, is included to highlight their critical aspects.

**Table 1:**
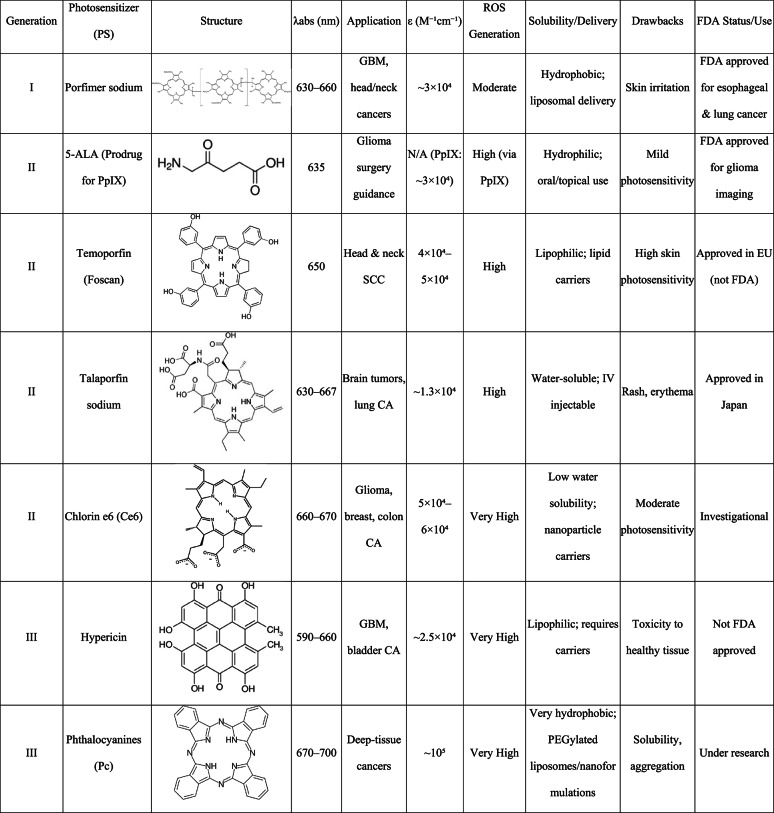
Classification of Photosensitizers and their Key Properties [56]

λ abs**:** Absorption peak wavelength; ε (M^-1^cm^-1^): Molar extinction coefficient, which measures the light absorption strength

#### First generation

The first generation of PSs consists of naturally occurring porphyrins such as hematoporphyrin. These PSs are characterized by their strong absorbance at approximately 400 nm; however, they have limited excitation potential at longer wavelengths [54]. Hematoporphyrin derivative (HpD) is composed of monomers, dimers and oligomers of hematoporphyrin. HpD is inefficient at producing singlet oxygen and requires prolonged exposure to light to obtain the desired therapeutic results [57].

#### Second generation

Second-generation PSs were developed to overcome the limitations of first-generation sensitizers. These PSs are activated by wavelengths greater than 600 and are comparatively more potent in singlet oxygen generation [58]. Chlorins and 5-aminolevulinic acid (5-ALA) are second-generation PSs. Among the commercially available PSs, 5-ALA is commonly used. When administered orally, it has a high bioavailability and safety profile. 5-ALA has strong selectivity for preferential accumulation in malignant gliomas and is relevant to GBMs [59]. 5-ALA is a porphyrin precursor that is essential for creating heme. Porphobilinogen is formed by the assembly of porphyrins and is converted into protoporphyrin IX (PpIX) by porphobilinogen deaminase. The expression of porphobilinogen deaminase is increased in glial tumor tissue, resulting in an increased amount of PpIX [60]. Typically, PpIX is converted to heme by the enzyme ferrochelatase. 5-ALA increases the concentration of PpIX in a GBM because of the decreased expression of ferrochelatase in the GBM, which decreases the conversion of PpIX to heme. PpIX absorbs blue light at 404 nm and emits fluorescence in the red spectrum, i.e., *at* 635 nm. PpIX is a PS that emits triplet oxygen and causes cytotoxicity when excited by 635 nm light.

#### Third generation

Third-generation PSs are known for their enhanced selectivity for targeting tumor cells. This improved selectivity is achieved via the use of modifiers, which include nanoparticles and antibodies. The development of these PSs has also focused on developing prodrugs that are explicitly activated by cancerous cells [61]. The main aim of designing third-generation PSs is to minimize off-target effects while optimizing the pharmacokinetics and properties of excitation and absorption. This approach aims to maximize the effectiveness of PDT while reducing side effects [62]. Common components that combine with PSs include monoclonal antibodies [63], saccharides [64], nanoparticles [65], hyaluronic acid [66], liposomes [67], polymer micelles [68], and small molecules [69]. Currently, no third-generation PSs are approved for use in PDT in humans.

### Therapeutic outcomes

Discussing preclinical and clinical aspects of medical research is vital for translating therapies from the laboratory to patient care. Preclinical studies assess safety and efficacy in animal models before human trials, identifying optimal dosing and potential side effects. Collaboration between researchers and clinicians enhances the predictive value of findings, leading to safer therapies and reducing attrition rates in early clinical trials.

Zhang et al. developed Sp-Exo/AI, which is composed of PLGA nanoparticles loaded with arsenic trioxide (ATO) and IR780 (NIR photosensitizer), which is further surface modified with GBM-derived exosomes and conjugated with SPIONs for magnetic targeting. ROS generation and photodynamic performance evaluations revealed that upon 808 nm laser irradiation, the SOSG fluorescence of SP-Exo/AI increased by 6.27x, which is a singlet oxygen indicator, suggesting a strong capacity for generating reactive oxygen species (ROS) for PDT. Similarly, the release profile of Sp-Exo/AI with a laser revealed 82% release after 24 h, whereas 65% release occurred without a laser, confirming the enhanced release of the Sp-Exo/AI. Additionally, the in vitro BBB model showed a fluorescence intensity 2.55x greater than that of free IR780 and 1.39x greater than that of PLGA/AI. The uptake was reduced to 0.59x with the membrane fusion inhibitor and 0.84x with the clathrin inhibitor, suggesting that membrane fusion was the dominant uptake route. The 3D model evaluations revealed that the penetration depth of free IR780 was approximately 20 μm, approximately 60 μm for Sp-Exo/AI, and 90 μm for Sp-Exo/AI + laser, along with apoptosis rates that were 1.41x and 1.55x greater than those of single drug-loaded vesicles. Moreover, ferroptosis activation was confirmed in the Sp-Exo/AI + L group, as indicated by a marked reduction in GSH levels, suppressed GPX4 activity, significantly elevated lipid peroxidation markers, and a 22.95-fold increase in ROS production compared with those in the PBS control group. Compared with Sp-Exo/IR780 + L, Sp-Exo/AI + L substantially induced immunogenic cell death, as implied by a 1.66x increase in CRT and a 1.48x increase in HMGB1 expression, with a marked increase in ATP release, dendritic cell mutation, T-cell activation, and increased secretion of IFN-γ and TNF-α. In vivo studies revealed 2.4x greater brain accumulation with magnetic guidance and an extended circulation half-life by 2.1x compared with free ATO+IR780. Moreover, liver and kidney accumulation was significantly reduced. In orthotopic glioma models and humanized PDX models, Sp-Exo/AI + L presented the strongest tumor suppression, with the lowest IVIS signal and an extended median survival of approximately 50% over that of saline. It also significantly reduced post-surgery recurrence when combined with TMZ and increased CD8 + T-cell infiltration and proinflammatory cytokine levels. The formulation is a highly promising platform for GBM therapy and immune checkpoint blockade (ICB) sensitization [70].

**Fig. 4:**
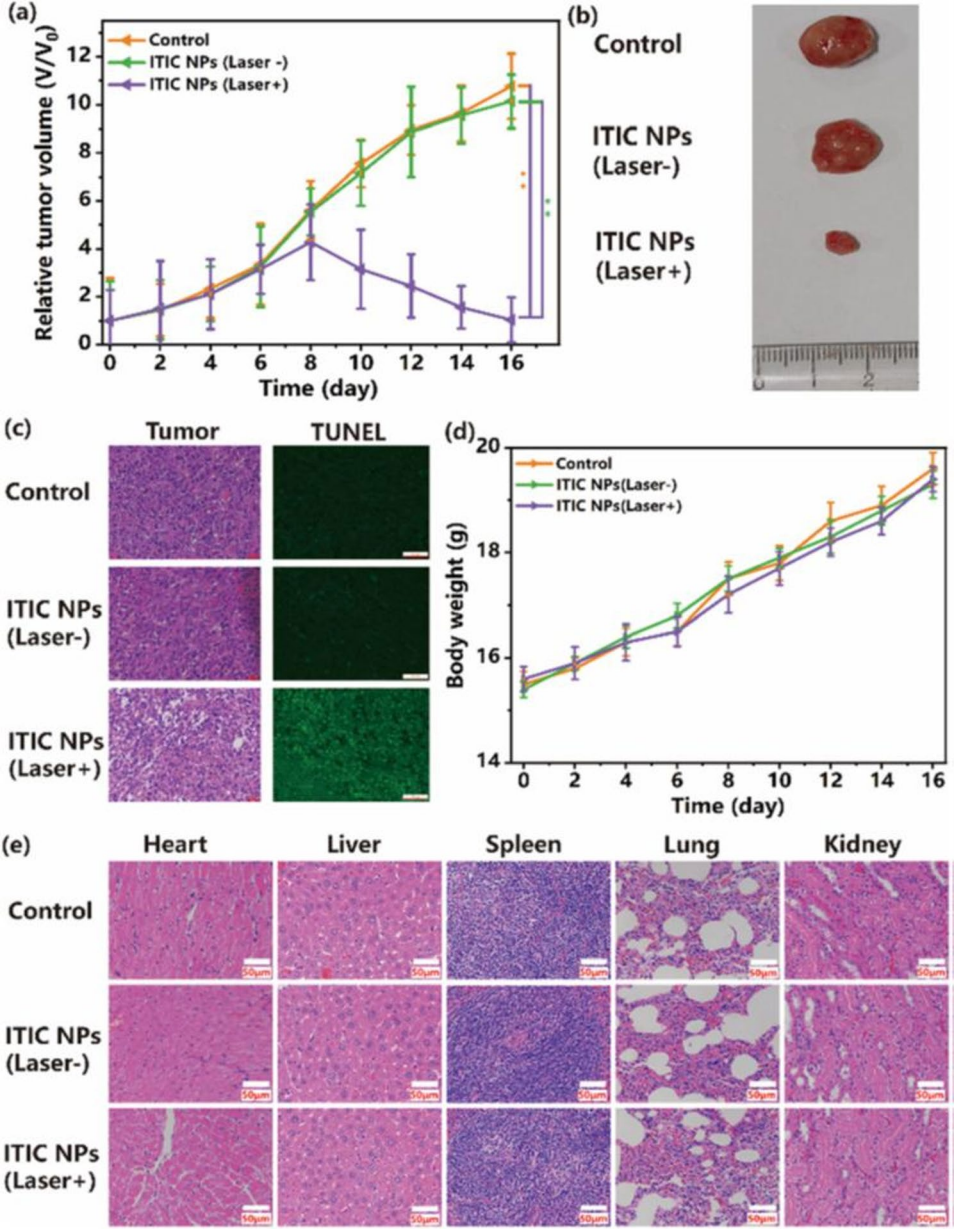
(**a**) Tumor volume changes in the mice. (**b**) Representative images of tumors. (**c**) H&E and TUNEL staining images of the tumor. (**d**) Body weight changes. (**e**) H&E images of the heart, liver, spleen, lung, and kidney. (Adapted with permission from [55])

A study by Liang et al. discussed the synthesis of a novel photosensitizer for laser-mediated photodynamic therapy of gliomas. ITICs were synthesized and developed into water-dispersible nanoparticles to increase the efficacy of treatment. The ITIC nanoparticles demonstrated excellent physicochemical characteristics, and the DSE-PEG2000 coating provided good dispersion and stability in physiological media. Similarly, optical characterization revealed a broad absorbance range with a redshifted peak at 739 nm for the ITIC NPs, suggesting strong NIR absorption, which is optimal for deep-tissue light penetration during PDT. The ROS generation efficiency was 10.27% when methylene blue was used as the standard, which was greater than that of BDPTPA and BDPA and was sufficient for effective PDT. The ITIC NPs showed a potent phototoxic effect with an IC_50_ of 7.08 µg/mL upon laser irradiation and minimal cytotoxicity in the absence of light, suggesting their light-specific activity. Confocal imaging confirmed strong intracellular ROS generation after NIR exposure, while Annexin V-FITC/PI and calcein-AM/PI staining significantly increased apoptosis and the highest degree of cell death in the ITIC NP + L group, confirming light-triggered cytotoxicity. Moreover, in vivo studies in nude mice, as shown in Fig. 4, revealed that while the tumor volume increased 10-fold in the control and no-laser groups, the ITIC NP + L group exhibited only a 1.2-fold increase in body weight, significant tumor cell apoptosis, and no observable damage to major organs, confirming both therapeutic and systemic biosafety. These findings suggest that ITIC NPs hold great promise for advancing PDT-based therapeutic strategies for GBM and potentially other deep-seated tumors [55].

**Fig. 5:**
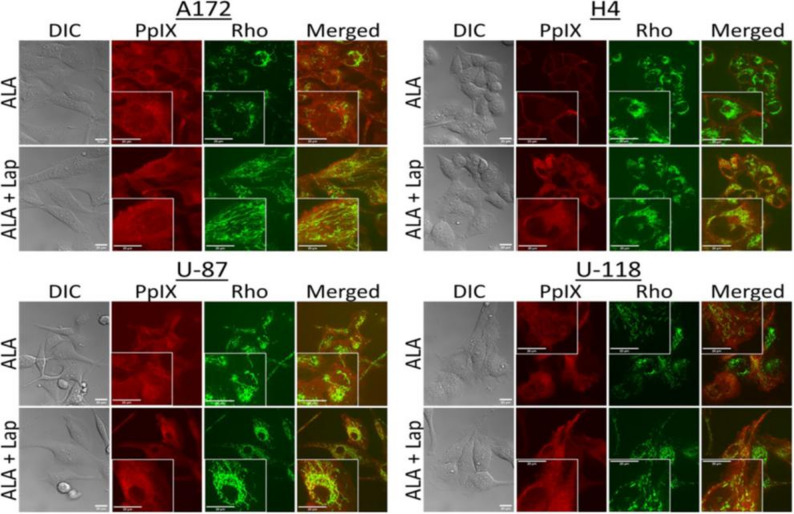
Intracellular localization of PpIX by confocal fluorescence microscopic imaging. GBM cells were treated with ALA (1 mM) alone or in combination with lapatinib (0.1 µM) for 4 h. Rhodamine 123 (Rho, 250 ng/mL) was added 30 min before imaging to label the mitochondria. The cells were imaged with a confocal fluorescence microscope to visualize the cell morphology via DIC, with PpIX fluorescence in red, Rho fluorescence in green, and merged images of PpIX and Rho fluorescence. Part of the image was magnified and is shown in the insert. Bars, 20 μm. (Adapted with permission from [71] )

Chandratre et al. developed 5-aminolevulinic acid-mediated PDT in combination with lapatinib to improve glioblastoma cell death by improving therapeutic efficacy through the mitochondrial localization of PpIX and the induction of apoptosis. ALA-PDT was found to reduce cell viability up to 80% in A172 and U-118 glioblastoma cell lines, but it did not have a significant cytotoxic effect on H4 and U-87 cells, suggesting variable sensitivity to PDT among different GBM models. In addition, lapatinib alone at a concentration of 0.1 µM did not reduce viability in the cell lines tested, indicating its lack of standalone toxicity. Moreover, the combination led to a significant reduction in cell viability among all the cell lines tested. Real-time monitoring of cell death over 48 h revealed that neither ALA-PDT nor lapatinib alone caused substantial apoptosis or necrosis in the cell lines. In contrast, the combination of the two significantly improved both apoptotic and necrotic signals in all four cell lines. The activation of apoptosis was confirmed by Western blot analysis, which revealed that the H4 cell line was the only one that exhibited cleavage of both PARP and caspase-9 following treatment, confirming the activation of the mitochondrial pathway to induce apoptosis. The results of confocal fluorescence microscopy depicted in Fig. 5 indicate that when ALA was employed alone, PpIX accumulation was weak and was limited to mainly the plasma membrane. However, when PpIX was used in combination with lapatinib, the fluorescence intensity of PpIX was substantially increased within the mitochondria of the cell lines. Similarly, quantitative colocalization analysis via Pearson’s correlation coefficients confirmed the increase in mitochondrial localization, with statistically significant increases, indicating that lapatinib promotes the retention and mitochondrial targeting of PpIX. The results of the Western blot analysis revealed that the H4 cell lines expressed no detectable Bcl-2 protein and low levels of the Bcl-xL protein, indicating that H4 cells were more sensitive than A172, U-87, and U-118 cells to treatment-induced apoptosis. On the other hand, A172, U-87, and U-118 cells expressed high levels of both Bcl-2 and Bcl-xL and therefore exhibited low levels of apoptosis induction, whereas all of the cell lines demonstrated equivalent accumulation of mitochondrial PpIX. Although lapatinib reduced EGFR phosphorylation in A172 and U-118 cells, it did not affect downstream S6 signaling, indicating that the enhancement of ALA-PDT is due mainly to ABCG2 inhibition and increased PpIX retention rather than suppression of EGFR-mediated survival pathways. These findings support lapatinib as a sensitizer of PDT-resistant GBM, suggesting a promising adjunctive strategy for apoptosis after surgery [71].

**Fig. 6:**
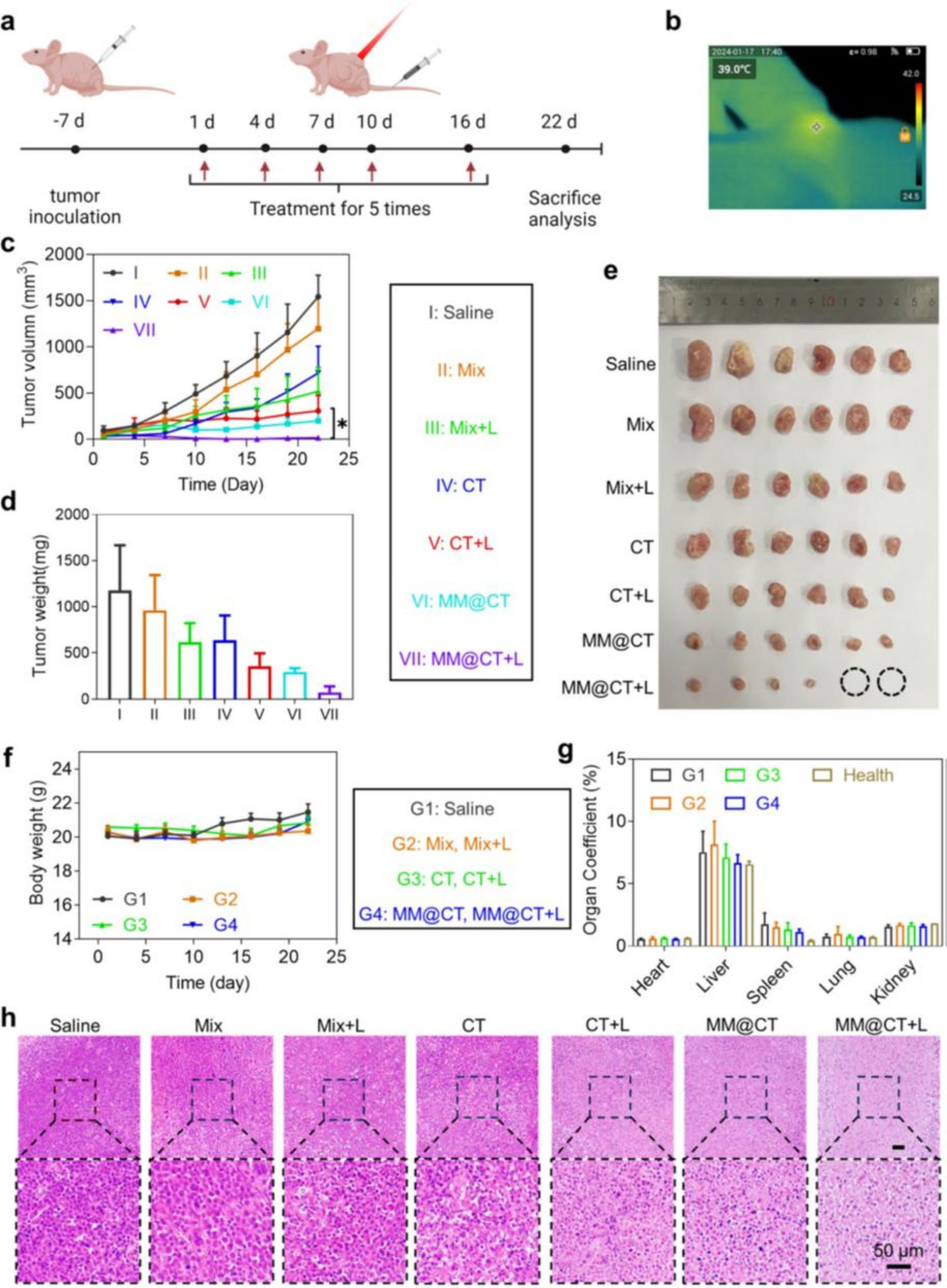
Anti-GBM efficacy in a subcutaneous tumor model. (**a**) A subcutaneous GBM tumor was established, with various treatment formulations applied. (**b**) Tumor site temperatures were maintained below 39 °C during phototherapy. (**c**) Tumor growth and (**d**) weight were monitored in the treatment groups. (**e**) Optical images of tumors were captured, and (**f**) the body weights of the mice were recorded. (**g**) Organ coefficients were evaluated, with healthy mice used as controls. (Adapted with permission from [72])

Cheng et al. developed a macrophage membrane-camouflaged nanomedicine (MM@CT) for synergistic treatment of glioblastoma. Researchers have evaluated the efficacy of the combination of chlorin e6 (Ce6) and temozolomide (TMZ) for the treatment of GBM via chemo-photodynamic therapy. The results of drug loading efficiency and characterization of the nanoparticles revealed an impressive 73.2% drug loading with a particle size under 100 nm and a zeta potential of -26 ± 0.58 mV. TEM revealed that the thickness of the macrophage membrane coating was approximately 12.5 nm. Similarly, the pH-responsive drug released 9.4% at pH 7.4 in 24 h, which was significantly greater than the 34.4% at pH 6.5 and 86% at pH 5.5. This indicated the stability of the nanoparticles in circulation and their targeted release at tumor sites. MM@CT also exhibited concentration-dependent singlet oxygen generation. Additionally, compared with that in the PBS control group, ROS generation in the 50 µM group was 3.4x greater. Moreover, the semiquantitative intracellular RPS of the MM@CT+laser group was 1.3x greater than that of the CT+laser group and 7.1x greater than that of the Mix+laser group. BBB penetration and cellular uptake studies revealed a 1.4x greater permeability in the in vitro BBB model than in the CT model. MM@CT also significantly enhanced internalization and lysosomal colocalization in U251 cells, suggesting efficient intracellular delivery, predominantly through clathrin-mediated endocytosis and, to a lesser extent, via caveolin and macropinocytosis. MM@CT demonstrated greater cytotoxicity both with and without laser irradiation. The combination index of TMZ and PDT when delivered via MM@CT was < 1, confirming its synergistic effect. The animal studies revealed that MM@CT resulted in increased tumor accumulation and reduced hepatic accumulation (0.54x greater than that of CT), indicating MPS evasion. Furthermore, in the orthotopic brain tumor model, the tumor fluorescence intensity was 4.9x greater, and the circulation half-life was 2.4x greater with MM@CT. The antitumor efficacy, as shown in Fig. 6, demonstrated complete tumor regression in almost 30% of the mice, with significant tumor volume and weight reduction in the MM@CT+laser group. Similarly, for biosafety evaluation, the hemolysis rate was found to be < 2% at 50 µM, confirming its blood compatibility. Thus, on the basis of these results, we can conclude that this approach has the potential for transforming glioblastoma treatment [72].

Table 1 provides a summary of preclinical trials comparing the efficacy of various PSs and treatment regimens in GBM therapy. The key parameters of each study, including the wavelength, concentration, dose of light, cell line, and in vivo performance measure, were used for comparison. Research has revealed a great diversity of conditions under which PSs can be used in the PDT of various cell lines in the human brain and preclinical models. The wavelength selectivity, optimal PS concentration, and suitable light dose differ from those of PSs to provide the highest therapeutic outcome. The data also shows that accurate dosimetry and controlled treatment conditions are essential for generating effective PDT results and minimizing possible off-target effects. Moreover, the versatile nature of PSs, such as Porfimer sodium, Temoporfin and Ce6, in attacking glioma is clear and paves the way for advancements in clinical phototherapy regimens. This highlights the importance of further research into optimizing these parameters to maximize treatment effectiveness and improve patient outcomes in cancer therapy.

**Table 2:** Overview of pathways, in vivo models, and outcomes of preclinical studies

Photosensitizer	Wavelength (nm)	Optical Concentration	Light Dose (J/cm²)	Cell Lines Studied	In vivo Studies	Outcomes	Ref
ALA (5-ALA) + Lapatinib	633 nm	1.0 mM ALA, 0.1 µM Lapatinib	3 J/cm²	A172, H4, U-87, U-118	Not mentioned	Significant viability reduction in all cell lines; H4 showed < 20% viability; apoptosis/necrosis increased; enhanced PpIX mitochondrial localization.	[71]
Verteporfin (VP)	689 nm	0.1–10 µM	18 J/cm² (30 s), 36 J/cm² (60 s)	LN229, HSR-GBM1, P1 (patient-derived)	No	VP-PDT caused significant cell death with EC₅₀ values as low as 0.22 µM (HSR-GBM1, 60 s); high VP uptake confirmed by flow cytometry.	[73]
5-ALA	Visible light (~ 600 nm)	Not stated	Not specified	Postsurgical GBM patients	Yes (retrospective cohort)	Inadvertent PDT from OR lights reduced recurrence within 1 cm of the surgical cavity (RR = 0.73, *p* = 0.017), supporting locoregional PDT effect.	[74]
Conjugated Polymer Nanoparticles (CPNs with PtOEP)	Blue light (460 ± 20 nm)	6–10 mg/L	10–40 J/cm²	MO59K, U-87 MG, T98G	GBM mouse xenograft model	CPN-PDT is effective in MO59K and U-87 MG; T98G was more resistant than the other two cell lines due to a higher level of antioxidant protein expression; mPDT was found to be superior to cPDT in terms of killing tumor cells with lower doses, being able to induce apoptosis, and slowing tumor growth in vivo studies.	[75]
PpIX (from ALA)	635 nm	25 µg/mL ALA	Up to 13 J/cm²	U251MG variants (EV, V)	No	ABCG2 overexpression reduced PpIX retention; inhibition restored PDT susceptibility; survival curves light-dose dependent	[76]
IR780-liposomal (targeted/nontargeted)	808	1.5 µg/mL	0.8–1 W/cm²	RG2, U87, L929	U87 tumor-bearing nude mice	Higher tumor uptake for targeted liposomes, lower toxicity in normal cells, effective PDT/PTT, and viable use for theranostic applications.	[77]
LN(AlClPc)Chitosan-loaded lipid system	630	0.0001% and 0.1% (w/w), 0.55 µmol/L	0.3 J/cm²	U87-MG, GL261	C57BL/6J mice (GL261 tumors)	Higher ROS production, strong tumor cell phototoxicity, decreased cell proliferation, and strong fluorescence imaging results.	[78]
Hypericin (HYP) in BSA-nanocomposite	Laser (not specified)	Not specified	Not specified	Not detailed, inferred GBM context	Yes (murine GBM model)	Enhanced ROS via ferroptosis and oxeiptosis pathways, targeting via SPARC, synergistic antitumor effects.	[79]
PANA (Polymeric Amino-Nano Aggregates)	Red emission (λ not specified)	Not specified	Not specified	Not specified	Mouse model	Strong PDT efficacy, GBM homing, high biosafety, and improved ISC for ROS generation.	[80]
Porphyrin-based MOF composites	Two-photon (λ not specified)	Not specified	Not specified	Not specified	Not specified	Improved penetration for deep tissue glioma, promising for gliolastoma PDT.	[42]

## Photothermal Therapy (PTT) in Glioblastoma

**Fig. 7:**
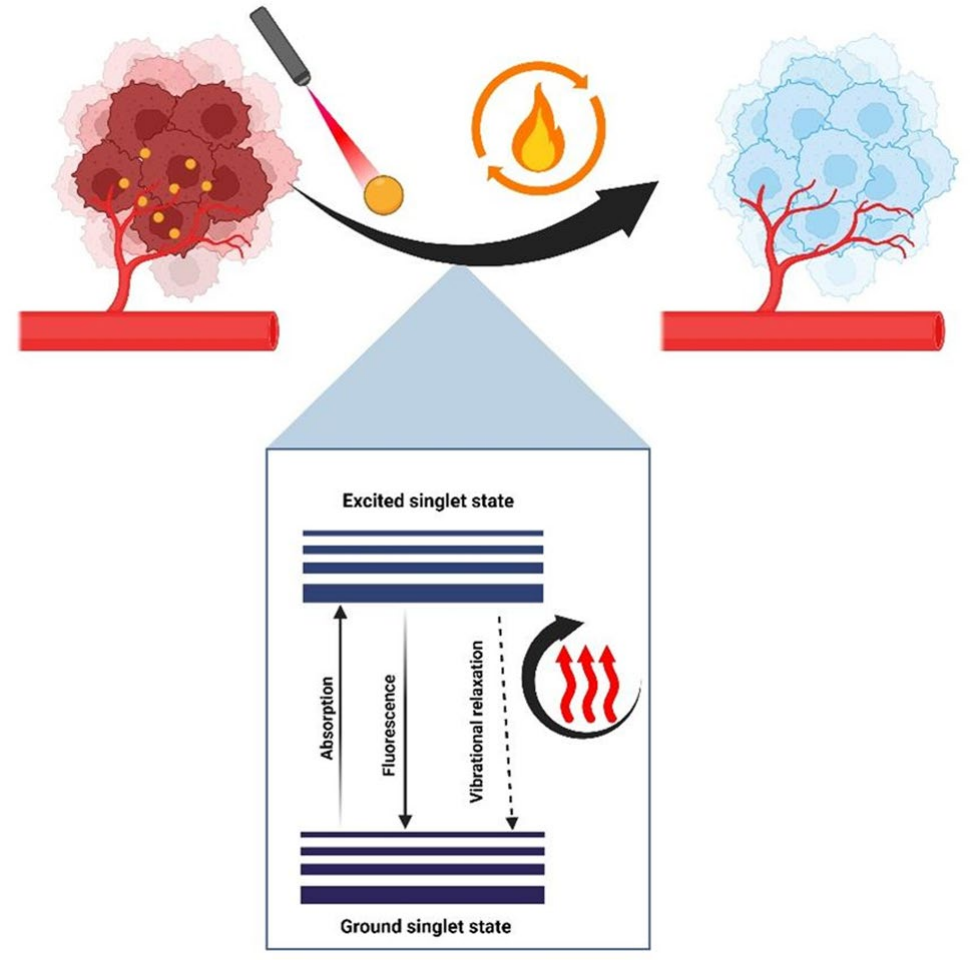
NIR-activated photothermal agents generate localized hyperthermia (> 42 °C), inducing apoptosis, necrosis, and immunogenic cell death in GBM while sparing healthy tissue. Created in BioRender. Dhas, N. (2025). https://BioRender.com/ncw9pax

PTT has high potential as a cancer treatment strategy because of its simplicity, short duration of therapy, rapid recovery, and noninvasive nature. PTT uses heat tolerance difference between malignant tissues i.e. ≥42 °C causes irreversible damage, and healthy tissues for precise thermal ablation as mentioned in Sect."Core Mechanisms of Phototherapy" and depicted in Fig. 7 [46]. Although PTT has independent therapeutic potential, it is frequently integrated with other treatments, such as chemotherapy, radiotherapy, PDT, gene therapy, and immunotherapy [50]. These multiple treatment modalities increase therapeutic effectiveness through improvements in drug delivery, reduced side effects, an enhanced immune response, and modifications of the TME. Nanomaterials are an important component of these combination therapies because they are capable of targeted delivery to cancer cells, sensitizing them to radiation therapy and modifying the immune response. This section discusses the advancements in synergistic combination therapies involving PTT, focusing on rationally designed nanocarriers that improve therapeutic delivery and efficacy.

Gold nanoparticles, carbon-based nanoparticles, polymer nanoparticles, and metal-organic frameworks serve as PTAs due to their unique optical, thermal, and biological properties [81]. These nanostructures concentrate in tumors via the enhanced permeability and retention (EPR) effect, with targeting ligands increasing affinity for tumor-specific biomarkers to reduce off-target effects [82].

### Types of photothermal agents (PTAs)

PTAs are important components of PTT in cancer therapy. These agents absorb NIR light and convert it to heat, efficiently targeting and destroying cancer cells. There are two main classes of PTAs: inorganic and organic materials. Inorganic PTAs include metal-, metal-oxide-, and carbon-based nanoparticles whose characteristics increase the efficacy of PTT [83, 84]. An example is gold nanorods and nanoshells, which have high photothermal conversion efficiencies but have biocompatibility and biodegradability problems. In contrast, organic PTAs such as polyaniline and polypyrrole can be used as possible biodegradable and biocompatible substitutes.

#### Inorganic PTAs

Inorganic PTAs have become a focal point in the advancement of PTT for cancer and other diseases owing to their robust optical properties and efficient light-to-heat conversion mechanisms [85]. These nanomaterials include not only metallic nanoparticles (e.g., gold and silver) but also metal‒oxide nanoparticles (e.g., iron oxide and copper oxide) and 2D nanosheets (e.g., black phosphorus), all of which are engineered to absorb NIR light that is highly penetrative into biological tissues and is safe [47]. The high photothermal conversion efficiency (PCE), structural stability, and easy functionality of these materials make them potential candidates for accurate, noninvasive ablation of tumors [86]. Moreover, inorganic PTAs are integrated with other therapeutic or diagnostic modalities, allowing the use of imaging-based therapies, drug therapies, and combination therapies. These agents are still considered the standard for performance and versatility in the PTT discipline, as research continues to face challenges related to long-term biocompatibility and systemic clearance.

##### Metallic nanoparticles

Photothermal therapy is built on the foundation of metallic nanoparticles because of their unique optical and electronic characteristics [87]. These nanoparticles, particularly those composed of noble metals such as gold and silver, exhibit strong surface plasmon resonance (SPR) effects when exposed to specific wavelengths of light, typically in the NIR region [88]. This SPR offers effective conversion of the photons absorbed into thermal power, which makes them powerful in the ablation of targeted and specific tumors. Their surfaces can also be easily functionalized with targeting molecules or therapeutic or imaging molecules, facilitating multimodal theranostic implementation.

Gold nanoparticles (AuNPs) constitute the gold standard in this category, with the advantages of being biocompatible, simple to prepare and having adjustable properties in terms of SPR [89]. They have a variety of morphologies, such as spheres, rods, stars, shells and wreaths, each with a different PCE [90]. For example, gold nanostars and nanorods exhibit strong NIR absorption, enabling effective deep-tissue PTT. AuNPs can also be functionalized with targeting ligands or drugs, enabling combined photothermal and chemotherapeutic or photodynamic effects [91]. Nanoparticles of silver and copper also exhibit SPR, but their long-term toxicity and stability remain a cause of concern [92]. Although less popular, rhodium and platinum nanoparticles offer distinctive catalytic and photothermal properties [93].

Metallic nanoparticles can be engineered for multimodal applications, including as PTAs, drug carriers, imaging contrast agents (MRI, CT, or photoacoustic imaging), and even radiosensitizers [94]. As an example, gold nano-wreaths with iron oxide nanoparticles demonstrate improved photothermal properties and enhanced imaging with MR images, whereas fluorescent gold nano-stars facilitate real-time monitoring of the effects of treatment [95].

##### Metal oxide nanoparticles

Metal oxide nanoparticles, such as iron oxide (Fe₃O₄), copper oxide (CuO), and titanium dioxide (TiO₂), represent another important class of inorganic PTAs [96]. Most of these materials, despite the absence of SPR, absorb NIR light and convert it to heat, thus rendering them useful in PTT.

Iron oxide nanoparticles (IONPs) are well known for their magnetic properties, which allow for magnetic targeting and MRI imaging. These materials have significantly improved photothermal efficiency when conjugated with gold or other metallic nanostructures. IONPs can be functionalized and have also been shown to be effective in PTT and radio-sensitization [97]. Copper sulfide (CuS) nanoparticles have good NIR absorption and PCE. They are less expensive than gold and can be synthesized in various forms, including hollow and core-shell structures, to maximize their therapeutic performance [98]. Titanium dioxide (TiO₂) nanoparticles are known primarily for their photocatalytic activity and have been used as sonosensitizers and photosensitizers in photodynamic and sonodynamic therapies [99]. However, TiO₂ can also be used to promote photothermal effects, especially when it is doped or mixed with other substances.

#### Organic PTAs

Organic PTAs represent a wide range of compounds, including small molecules, polymers, and carbon-based nanomaterials, that absorb light, usually in the NIR, and transform it into heat to ablate tumors [83]. The main benefits of these materials are their biocompatibility, biodegradability and versatility in chemistry.

π-Conjugated polymers are among the most popular organic PTAs. These materials, such as polyaniline, poly-pyrrole, and polythiophene derivatives, possess extended conjugated systems that enable strong NIR absorption and optimal PCE [100]. Researchers have developed π-conjugated polymer nanoparticles with excellent optical properties that can produce high temperatures with NIR irradiation and can successfully destroy brain tumor tissue in preclinical trials. Several organic PTAs, such as indocyanine green (ICG), IR-780, and other cyanine derivatives, are extensively used as small-molecule dyes [101]. These dyes have high NIR absorption, are FDA-approved for some uses (e.g., ICG as an imaging agent), can be incorporated readily into nanoparticles, or can be conjugated to them to allow targeted delivery to samples. They also often have drawbacks of quick photobleaching and short circulation times, and encapsulation by liposomes or polymeric carriers is a modality that is currently being explored to counteract these drawbacks [102]. Carbon-based nanomaterials, including carbon dots, graphene oxide, and carbon nanotubes, exhibit excellent absorption of NIR light and a high photothermal conversion rate [103]. Carbon dots, for example, have been shown to generate significant heat under NIR irradiation and can be engineered for tumor targeting and imaging [104]. Graphene oxide and carbon nanotubes are also under investigation because of their unique optical, electrical and mechanical characteristics despite the lack of understanding of their long-term toxicity [105]. An emerging category of PTA consisting of both organic and inorganic constituents is metal‒organic frameworks (MOFs) and organic‒inorganic hybrid materials, which combine the benefits of both inorganic and organic constituents. π-Conjugated MOFs have been demonstrated to be very effective as tumor ablation agents in preclinical brain tumors [106].

In conclusion, the PTA landscape is evolving very quickly, with both inorganic and organic materials presenting specific benefits in cancer treatment. Inorganic PTAs such as metallic and metal‒oxide nanoparticles remain of great interest because of their high photothermal conversion efficiency, structural flexibility, and usefulness in multifunctional theranostics. Moreover, research into organic PTAs, including π-conjugated polymers, small-molecule dyes such as indocyanine green and IR780, diketopyrrolopyrroles, and croconaine-based agents, has opened up the horizons of safer, more biocompatible and tunable photothermal therapies. To offer a broad perspective, we have also explained the related case studies that point to clinical and preclinical advancements in the field. Additionally, we have added a detailed table, Table 1, which summarizes different types of PTAs and their main characteristics, thus providing a clear comparison to guide future research and application.

**Table 3:** Types of photothermal agents. Adapted with permission from [107]

Nanomaterial	Size	Laser irradiation	Photothermal effect	Properties
**Inorganic nanomaterials**				
Metallic nanomaterials				
Au nanostructures	10–50 nm	528–808 nm 500mW cm^− 2^– 4 W cm^− 2^ 4–20 min	ΔT increases up to 30 °C Temperature increases up to 55 °C	High PCE Varied Cytotoxicity Effective tumor ablation
CuS Nanoparticles	1–20 nm	980 nm 1.5 W cm^− 2^ 10 min	Temperature increases up to 53 °C	
Pt Nanoparticles	1–20 nm	808 nm 1.2 W cm^− 2^ 10 min	ΔT increases up to 65 °C	
Metal oxide nanomaterials				
Fe_3_O_4_ Nanoparticles	Up to 80 nm	808 nm 3 W cm^− 2^ 500 s	Temperature increases up to 63 °C	Excellent electron transport Plasmon resonance-dependent heat conversion Efficient reduction in tumor growth
Carbon-based nanomaterials				
Carbon Nanotubes	50–500 nm	980 nm 2 W cm^− 2^ 20 min	Temperature increases up to 45 °C	Strong light absorption Good thermal stability
Graphene Oxide (GOs)	200–2000 nm	808 nm 1 W cm-2 5 min	Temperature increases up to 50 °C	Excellent light absorption Tunable properties through functionalization Low thermal conductivity
Reduced Graphene Oxide (rGO)	1–5 μm	980 nm 2 W cm-2 15 min	Temperature increases up to 70 °C	High surface area Excellent conductivity
**Organic nanomaterials**				
ICG	10–200 nm	808 nm 1 W cm^− 2^ 8 min	ΔT increases up to 15 °C Temperature increases up to 53 °C	Strong absorption and fluorescence in the NIR region Better deep tissue penetration
Phthalocyanine	6–350 nm	670–808 nm 1.5–6.4 W cm^− 2^ 2–10 min	ΔT increases by 30 °C	Thermally stable Exhibit strong light absorption in the visible spectrum
Cryptocyanine	1 to 100 nm	730 nm 3 W cm^− 2^ 5 min	Temperature increases by 25 °C	High Surface Area-to-Volume Ratio Exhibit quantum mechanical behaviors
Diketopyrrolopyrrole	50–200 nm	660 nm 0.2–1 W cm^− 2^ 5 min	ΔT increases by 20 °C	Excellent photostability
Croconaine	10–100 nm	780–830 nm 3.5 W cm^− 2^ 10–20 min	Temperature increases by 45 °C	High PCE Excellent Photostability
Porphyrin	20–200 nm	630–808 nm 1–2 W cm^− 2^ 10 min	ΔT increases by 20 °C	Enhanced catalytic activity
Polyaniline	50–600 nm	808 nm 1–2 W cm^− 2^ 3–5 min	Temperature increases by 40 °C	Exhibits higher conductivity
Polydopamine	10–900 nm	808 nm 0.8 W cm^− 2^ 9 min	ΔT increases by 19 °C	Enhanced cellular uptake Improved antioxidant properties

### Therapeutic outcomes

Having established the fundamental principles and mechanisms of PTT, we focus on its therapeutic outcomes, which have been demonstrated in preclinical and clinical studies. Various investigations have reported promising outcomes, highlighting the potential of PTT to selectively ablate tumor tissue, reduce recurrence rates, and improve patient prognosis when integrated with other modalities. The following case studies illustrate the diverse applications of PTT across glioblastomas and its treatment settings. Furthermore, to provide a comprehensive overview, a summary table, Table 3, stating the tumor models, cell lines and other key therapeutic outcomes, is also included.

Liu et al. synthesized and developed an exosome‒liposome hybrid-based theranostic nano-system for NIR-II fluorescence imaging-guided and targeted PTT for subcutaneous glioblastoma. C12 and NIR-C12, cyanine dyes, were synthesized, with NIR-C12 exhibiting a redshifted absorbance at 1045 nm and emission at 1101 nm due to a reduced bandgap, which was confirmed by DFT modeling. These materials were fabricated via lipid film hydration and extrusion techniques; compared with the NIR-C12-L hybrid, the exosome-liposome hybrid NIR-C12-EL demonstrated stable membrane fusion, intermediate zeta potential, and superior photostability and a fluorescence quantum yield of approximately 0.781%, whereas the NIR-C12-L hybrid yielded approximately 0.577%. Moreover, for photothermal evaluation, the temperature of the NIR-C12-EL rapidly increased to 78.2 °C within 9 min under a 1064 nm laser (1 W/cm^2^) at 1 mg/mL, achieving a high PCE of 62.28% and retaining approximately 82% of the absorbance after 10 heating/cooling cycles. In vitro analysis revealed that NIR-C12-EL had superior cellular uptake ability and biocompatibility, with significant tumor cell death observed only when combined with laser irradiation. The live/dead assay results revealed strong red fluorescence, which suggested extensive cell death in the NIR-C12-EL+laser group compared with the NIR-C12-L+laser group, which resulted in partial killing. In vivo fluorescence imaging and biodistribution revealed peak tumor fluorescence after a 2 mg/kg dose of NIR-C12-EL at 12 h postinjection, along with higher intensity and tumor accumulation than those of NIR-C12-L, which was primarily localized to the liver and spleen. Similarly, in vivo PTT revealed that 5 min of laser irradiation elevated the tumor temperature to 55 °C in the NIR-C12-EL+laser group, leading to extensive tumor necrosis, whereas 48.3 °C in the NIR-C12-L+laser group resulted in partial necrosis. Moreover, tumor growth was completely inhibited after NIR-C12-EL+laser irradiation and 100% survival was achieved on day 60, whereas in the NIR-C12-L+laser and control groups, tumor relapse and 30% survival by days 40–45 were observed, with no weight loss or organ toxicity. These findings support the potential of NIR-C12-EL as a highly effective nano-theranostic agent for NIR-II image-guided photothermal GBM therapy [108].

**Fig. 8:**
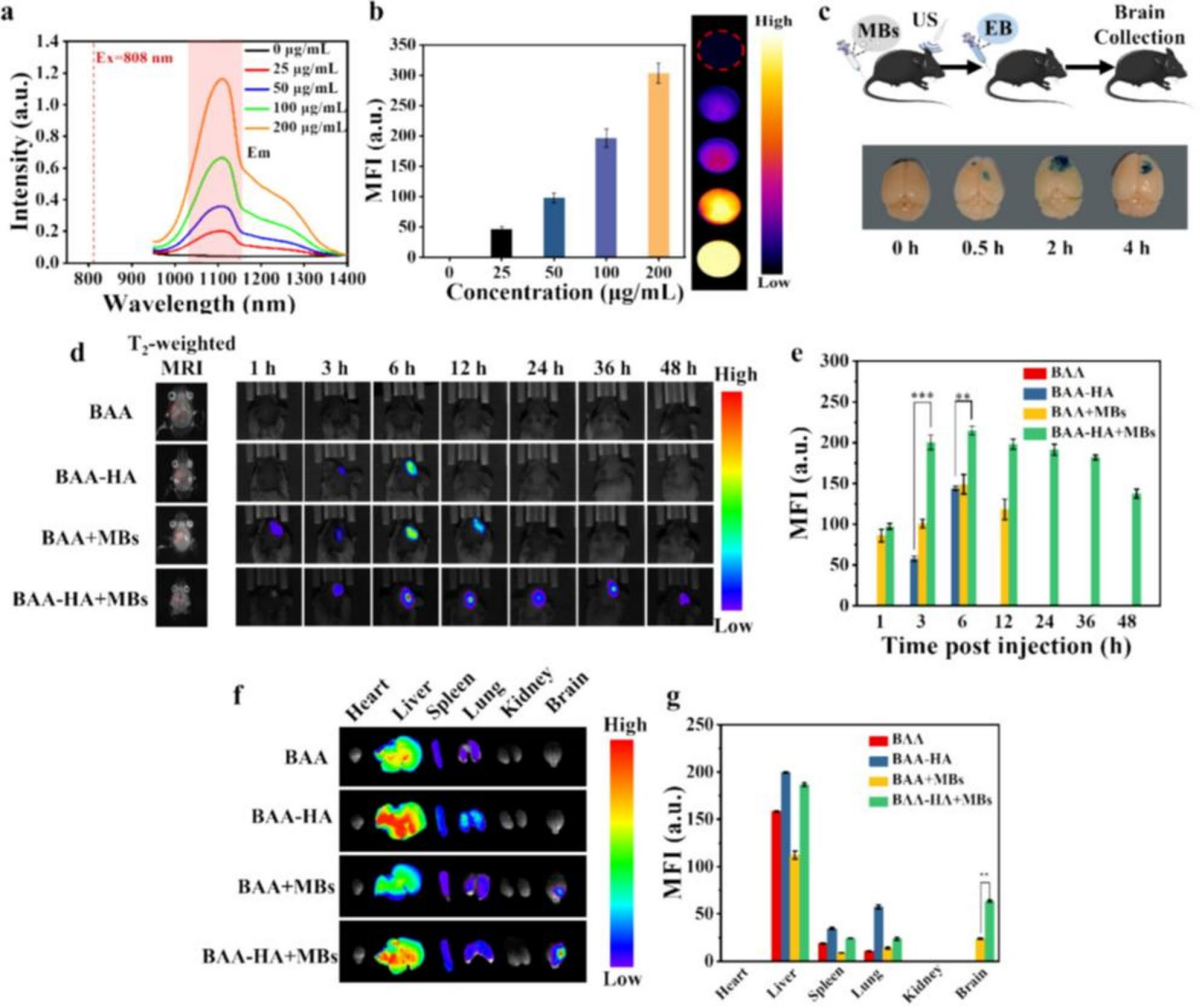
In vivo BBB opening and NIR-II fluorescence imaging of BAA-HA. (**a**) Fluorescence spectra of BAA-HA at various concentrations under 808 nm laser excitation. (**b**) Quantitative fluorescence intensity of different concentrations of BAA-HA under 808 nm laser excitation. (**c**) Schematic representation of US- and MB-mediated BBB opening in vivo. (**d**) MR images and real-time NIR-II fluorescence images of nanoparticle accumulation at the tumor site after various treatments with an 808 nm laser. (**e**) Quantitative analysis of fluorescence intensity at the tumor site over time. (**f**) Ex vivo fluorescence imaging of major organs from mice under an 808 nm laser at 6 h posttreatment. (**g**) Quantitative fluorescence intensity of major organs. (Adapted with permission from [109])

Qu et al. developed and evaluated multifunctional FePt-based SPANs for multimodal GBM therapy. They developed a nanoplatform, Boron nanosheets-Gold-Silver Sulfide, conjugated with hyaluronic acid for CD44 targeting, with a diameter of approximately 120 nm, a zeta potential of -21 mV, and an AFM thickness of 3.5 nm, as confirmed by X-ray diffraction (XRD), Fourier transform infrared (FTIR), and X-ray photoelectron spectroscopy (XPS). For photothermal and sonodynamic performance evaluation, BAA-HA increased the temperature by 38.9 °C in 10 min, with a PCE of 46%. Furthermore, when combined with ultrasound, it achieved almost 90% DPBE degradation, a 1.8-fold increase in singlet oxygen generation. For enzyme-mimicking catalytic activity, BAA-HA presented a 1.7x faster glucose consumption and O_2_ generation rate of 0.28 mM/min, indicating enhanced Gox-like and CAT-like catalytic activities. An increase in BBB permeability of 3.2-fold was observed with ultrasound-microbubble treatment, and in vivo NIR-II imaging revealed that tumor accumulation peaked at 8 h postinjection, with 2.5x increased fluorescence compared with that of the controls. Additionally, the in vitro cytotoxicity studies in U87 cells revealed almost 90% cell death for BAA-HA + US + Laser compared with 68.1% for BAA-HA + US, 56% for BAA-HA + Laser, and < 10% for the control groups, along with 3.3x greater CRT exposure and 2.8x greater HMGB1 release than the control. In the orthotopic GBM model, the tumor volume was found to be reduced by 91% for BAA-HA + laser + US, 68% for BAA-HA + US, 53% for BAA-HA + laser, and 3.4% for the control. Similarly, the median survival was found to have increased to 46 days, with an increase in the levels of IFN-γ, TNF-α, and IL-6 of 2‒3x, as shown in Fig. 8. Biosafety evaluation revealed no significant changes in functional markers or organ toxicity, confirming good biosafety [109].

Bybordi et al. worked on gold/platinum nanorods and a TMZ-UiO-66-NH_2_ MOF loaded in chitosan-grafted PCL/PCL core-shell nanofibers for GBM treatment during chemo-photothermal therapy. The TMZ encapsulation efficiency was highest in the core-shell nanofibers, i.e., approximately 98%, which decreased to 84% after Pt-Au coating, suggesting efficient but reduced loading due to surface modification. The release profile of TMZ from the Pt-Au-coated nanofibers extended for up to 62 days at pH 7.4 and 58 days at pH 5, which was reduced to 44 and 36 days under NIR irradiation. In terms of photothermal activity, the temperature increased to 44.5 °C only for the Pt-Au-coated nanofibers, which exceeded the threshold for efficient tumor ablation. Furthermore, the viability of U87 cells decreased to 20% after NIR irradiation of the Pt-Au-coated nanofibers, whereas it was 57% without Pt-Au. Moreover, apoptosis markers increased Bax, CASP-3, and CASP-9 and downregulated Bcl-2 expression in treated U87 cells, indicating improved apoptotic activity. For in vivo studies, mice treated with TMZ-Pt-Au-MOF-core-shell nanofibers under NIR irradiation presented the lowest tumor volume of 0.48 V/V_0_ after 30 days. Simultaneously, biocompatibility confirmed that the treated groups maintained stable body weights, and the viability of normal cells was > 90%. This study successfully demonstrated a synergistic chemo-photothermal therapy system, paving the way for personalized medicine [110].

**Table 4:** Therapeutic outcomes of photothermal therapy for brain cancer

Photothermal Agent Used	Size	Laser Irradiation	Cell Line Studies	In vivo Studies	Outcomes	Ref
IR783 + CPT-RT	~ 150 nm (DLS), ~ 120 nm (TEM)	808 nm, 0.8 W/cm², 5 min	LN229	Nude mice (Luc-LN229 orthotopic model)	Tumor inhibition, extended survival, low systemic toxicity	[111]
ICG (in microbubbles)	~ 120–130 nm (DLS)	808 nm, 1.5 W/cm², 500 s	U87 MG, C6, GL261	Nude mice (orthotopic glioma)	Enhanced BBB penetration, rapid accumulation, tumor temp ~ 64.5 °C	[112]
Cu₉S₈ (NIR-II PTT)	161 nm (TEM); 188.6 ± 12.5 nm (DLS)	NIR-II (exact nm not specified)	LN229	Orthotopic glioma in mice	Synergistic CDT/PTT/chemo/starvation, efficient BBB crossing	[113]
ICG + MCT4-antibody	Not specified	NIR-II (1064 nm), 5 min	Not mentioned	Orthotopic mouse model	Clear tumor margins, no residual cells, survival benefit	[114]
Fe₃O₄@SiO₂@PLTM + TMZ	~ 150–160 nm	808 nm, 1 W/cm², 10 min	GBM cells (unspecified)	In vitro only	80% cell death at 65 °C, MRI-visible, pH-responsive release	[115]
Graphdiyne (GDY) + FIN56	Not specified	808 nm, 2 W/cm², 5 min	LN229, T98G	Orthotopic xenograft mouse model	Ferroptosis + PTT synergy, prolonged survival, BBB penetration	[116]
ICG + cRGD peptide	Not specified	1064 nm, 1 W/cm², 5 min	U87 MG, C6, GL261	C57BL/6 mice (GL261 orthotopic model)	Tumor temperature ~ 45 °C, effective PTT, high biosafety	[117]
TMZ-conjugated GNPs	~ 45.88 ± 1.9 nm	Not specified	T98G	Nude mice, subcutaneous GBM	Enhanced uptake, MGMT downregulation, prolonged survival (46 days)	[118]

## Biomedical applications of the intranasal route of administration

The intranasal route of drug delivery has emerged as a noninvasive and BBB-bypassing approach for brain-targeted therapies. This text presents innovations in intranasal formulations for glioblastoma treatment, including thermally responsive nanocarriers and antibody-conjugated systems. Preclinical studies have also combined intranasal delivery with targeted, stimuli-responsive nanoplatforms to enhance therapy (Table 4).

**Fig. 9:**
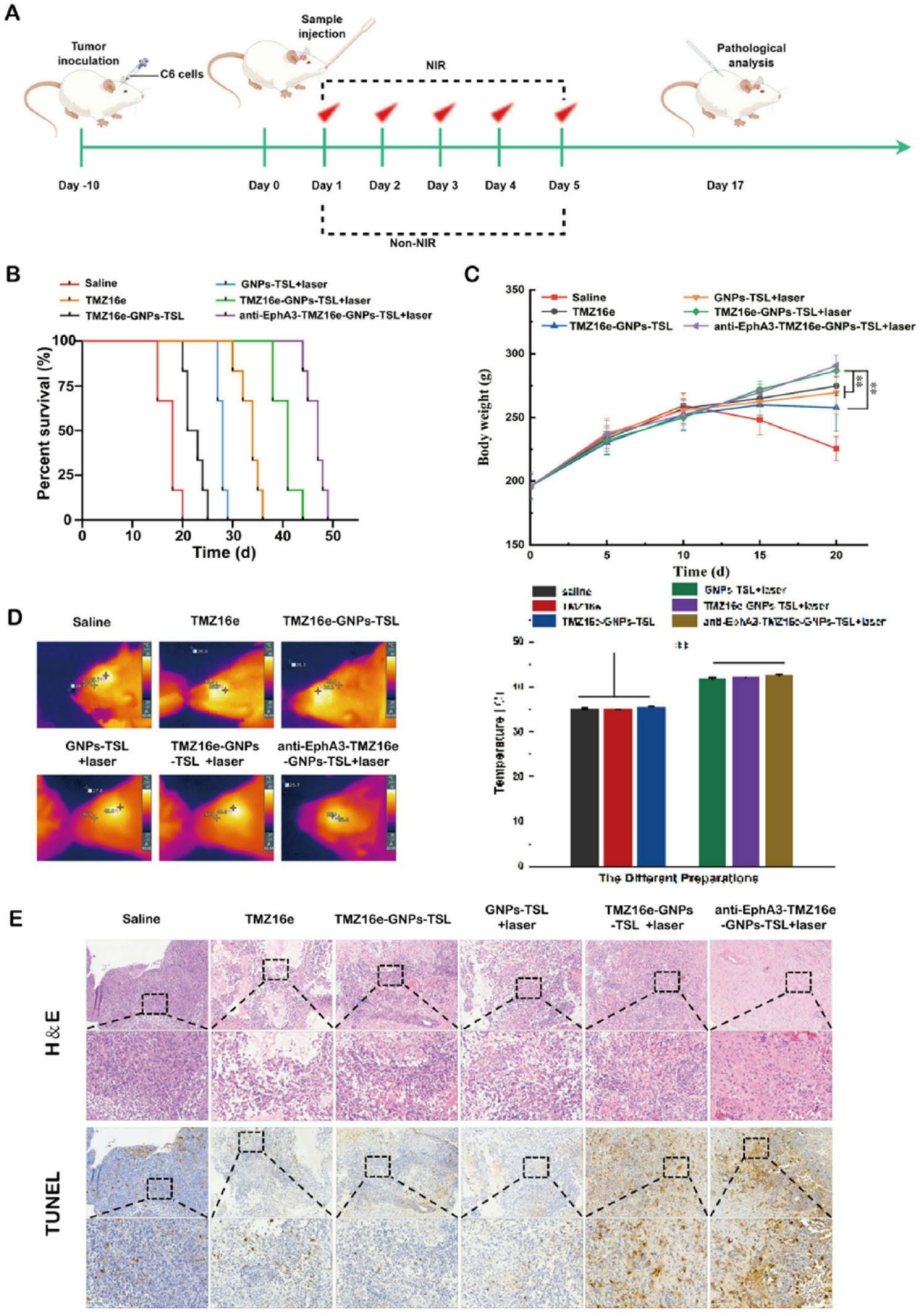
In vivo efficacy assessment. (**A**) A schematic illustration of the treatment protocol. (**B**) Kaplan‒Meier survival curves for tumor-bearing rats in the treatment groups. (**C**) Changes in body weight of the tumor-bearing rats in the treatment groups. (**D**) Photothermal imaging of the rat brain and the effect of photothermal action in vivo. (**E**) H&E and TUNEL staining of tumor tissue. (Adapted with permission from [119])

Duan et al. developed thermosensitive liposomes loaded with gold nanoparticles and TMZ hexadecanoate for a targeted synergistic approach for glioblastoma therapy [119]. This nano-formulation, an anti-EphA3-modified thermosensitive nano-system, was delivered intranasally. The developed nanoparticles had a uniform size of approximately 170 nm, a zeta potential of -25 mV, a PDI of < 0.2, and a phase transition temperature of 42 °C, with encapsulation efficiencies of approximately 5% for TMZ16e and 80% for GNPs and an antibody conjugation efficiency of approximately 25%. The results of in vitro drug release showed that TMZ16e-GNP-TSL released 81% of the drug within 2 h at 42 °C after NIR irradiation, whereas only 20% of the drug was released at 37 °C, suggesting strong thermal responsiveness. The evaluation of the photothermal properties revealed that the developed nanoparticles consistently achieved temperatures above 42 °C, which is adequate for inducing drug release, under 780 nm NIR irradiation at 2 W/cm² for 5 min. It was also found to demonstrate excellent thermal cycling stability across five repeated heating/cooling cycles. Nasal compatibility was confirmed in HNEpC cells, which were found to maintain over 90% viability after 2, 4, and 6 h of exposure to TMZ16e-GNPs-TSL and anti-EphA3-TMZ16e-GNPs-TSL. Similarly, the highest cellular uptake efficiency was demonstrated by nanoparticles conjugated with a laser, resulting in the highest cellular uptake and significantly higher fluorescence intensity than those of the unmodified TSL. Moreover, pretreatment with anti-EphA3 antibodies reduced cellular uptake, indicating receptor-specific targeting. The results of in vivo anti-GBM efficacy, as depicted in Fig. 9, revealed that the tumor site temperature increased by 6.4 °C in the conjugated nanoparticle + laser-treated group, in which the longest median survival time of 47 days was observed, compared with 41 days in the nonconjugated + laser-treated group, 34 days in the TMZ16e-treated group, and 28 days in the GNP-TSL + laser-treated group, with stable body weights indicating minimal systemic toxicity. Additionally, TUNEL and H&E staining confirmed the most extensive tumor apoptosis and necrosis in the conjugated nanoparticles + laser group. Histological analysis of the major organs revealed no signs of damage in any group, and the temperatures posttreatment returned to baseline within 24 h, suggesting effective clearance and no thermal accumulation. These results showed that the approach is safe and effective, suggesting that it is a clinically relevant strategy for noninvasive glioblastoma treatment.

**Table 5:** Preclinical studies of targeted photothermal–chemotherapy and intranasal nanoplatforms for glioblastoma

Main components	Targeting moiety / Delivery route	Light wavelength & PTT conditions	Combination modality	Model	Key findings	Survival/Tumor inhibition	Ref.
Temozolomide (TMZ)-conjugated gold nanoparticles (GNPs)	Anti-EphA3 antibody	808 nm NIR, concentration- and time-dependent (ΔT up to 47 °C at 200 µM)	Chemo-photothermal therapy (overcomes TMZ resistance)	TMZ-resistant T98G cells; subcutaneous T98G xenograft (nude mice); intranasal feasibility in rats	IC₅₀ reduced from 1480 µM (free TMZ) to 5 µM (anti-EphA3-TMZ@GNPs + NIR) (> 281-fold enhancement) Apoptosis rate 67.5% (targeted + NIR) vs. 9.8% (TMZ alone) G2/M arrest only in PTT groups Downregulation of MGMT and Bcl-2, upregulation of p53, Bax, p21 Tumor inhibition: 88% (targeted + laser) vs. 11% (TMZ alone) • Intranasal route achieved brain ΔT = 40 °C vs. 35.1 °C (IV)	Tumor growth inhibition 88% (subcutaneous model); intranasal superior to IV for brain heating	[120]
FePt nanoparticles loaded with lenalidomide (LND); surface-modified with hyaluronic acid + lactoferrin (SPANs)	Hyaluronic acid (CD44) + lactoferrin/Intranasal administration	Not applied in vivo (in vitro magnetic hyperthermia + Fenton ROS only)	Chemo + magnetic hyperthermia + Fenton reaction + ROS generation	U87MG cells; ex vivo nasal mucosa; in vitro BBB model; healthy rat brain (intranasal pharmacokinetics)	pH-responsive release (> 90% at pH 5.5, < 15% at pH 7.4) High cellular uptake (26.8 pg Fe/cell at 48 h); clathrin/caveolae-mediated + CD44 targeting Strong intracellular ROS via Fe leaching/Fenton Cell viability reduced to ~ 54% at 10 µg/mL • Rapid brain delivery (peak at 60 min post-IN); excellent mucus penetration and BBB crossing without toxicity	Only pharmacokinetic & safety study performed, highly efficient nose-to-brain delivery confirmed	[25]

All these studies confirm that the intranasal route is an efficient and convenient approach for administering phototherapeutic and chemotherapeutic agents to brain tissues. It has potential for clinical translation, especially when used in conjunction with image-guided phototherapy systems.

## Clinical trials

Clinical trials are an essential step in translating promising results of preclinical research to practical clinical applications in GBM. With photodynamic and photothermal therapies showing promising results in laboratory and animal experiments, several clinical trials have been launched to determine their safety, dosage studies, and efficacy in humans. These clinical trials evaluate single and combination regimens of phototherapy, using a variety of PSs and PTAs along with routes of light delivery in a variety of patient groups. The section below summarizes major currently running and already completed clinical trials that have focused on phototherapy-based interventions for GBM and their aims, the specifics of the interventions, the phases involved, and their results where possible. These insights can be critical in assessing the clinical preparedness and future effects of light-based multimodal therapies in the glioblastoma treatment paradigm. Table 5 provides information on clinical trials evaluating phototherapy in GBM treatment.

## Patents

The growing interest in phototherapy for GBM has driven an influx of new technologies, most of which are covered by intellectual property applications. Patents are an invaluable part of research expansion and commercialization, as they protect innovative formulations, light-delivery systems, photosensitizers, and drug-delivery/target-delivery systems. The development of next-generation phototherapeutic platforms, ranging from nanoparticle-based agents to hybrid diagnostic-therapeutic devices, has been actively documented through patent applications. Table 6 provides a curated overview of key patents relevant to phototherapy in GBM, reflecting the technological advancements and translational focus in this emerging therapeutic domain.

**Table 6:** Clinical trials related to phototherapy for GBM treatment

Study Title	Intervention/Treatment	Purpose	Status	Clinical Trials.gov Identifier	Ref.
5-Aminolevulinic Acid (5-ALA) Gliolan^®^: Usage Increase Proposal for Neurosurgical Procedures in High-Grade Gliomas	5-ALA	To assess disease-free survival (DFS) in patients with malignant gliomas undergoing neurosurgical procedures using 5-ALA-based PDT	Observational	NCT05850377	[59]
INtraoperative photoDYnamic Therapy of GliOblastoma (INDYGO)	5-ALA- PpIX	To evaluate the feasibility of a “5-ALA- PpIX (protoporhyrin IX) mediated per-PDT protocol” in patients with glioblastoma in addition to the stupp protocol.	Completed	NCT03048240	[121]
Photodynamic Therapy With Porfimer Sodium in Treating Patients With Refractory Brain Tumors	Porfimer sodium	To examine the side effects and best dose of PDT using porfimer sodium in treating patients with refractory brain tumors comprising astrocytoma, ependymoma, and medulloblastoma.	Phase I	NCT00002647	[122]
High Light and Low Light Dose PDT in Glioma	Porfimer sodium	To assess two different light doses of PDT using porfimer sodium to compare how well they work in treating patients undergoing surgery for recurrent malignant astrocytoma.	Completed	NCT00118222	[123]
Photodynamic Therapy (PDT) for Recurrent Pediatric Brain Tumors	Porfimer sodium	The study aimed to evaluate a new PDT modification that could revolutionize the treatment of brain tumors in children and adults.	Completed	NCT01682746	[124]
Intracavitary Photodynamic Therapy as an Adjuvant to Resection of Glioblastoma or Gliosarcoma Using IV Photobac^®^	3-(1-Butyloxy)ethyl-3-deacetyl-bacteriopurpurin-18-n-butylimide methyl ester	To evaluate the safety of this photosensitizing drug, an eight-step dose escalation will involve three patients per step.	Phase 1	NCT05363826	[125]
Magnetic Resonance Imaging-Guided Laser Induced Thermal Therapy for Treatment of Metastatic Brain Tumors	MRI-Guided Laser Induced Thermal Therapy	The purpose of this study is to evaluate the feasibility and safety of using MRI-guided laser-induced thermal therapy (LITT) for treating metastatic brain tumors that cannot be removed by surgery.	Phase 2	NCT00787982	[126]
Surgery, Radiation Therapy, and Chemotherapy With or Without Photodynamic Therapy in Treating Patients With Newly Diagnosed or Recurrent Malignant Supratentorial Gliomas	Porfimer sodium	The purpose of this study is to determine whether adding PDTto standard treatment improves time to recurrence and survival in patients with newly diagnosed or recurrent malignant supratentorial gliomas.	Phase 3	NCT00003788	[127]
LITT Followed by Hypofractionated RT for Newly Diagnosed Gliomas (GCC 20138)	LITT followed by Hypofractionated Radiation Therapy	The aim of this study is to assess the effectiveness and safety of combining Laser Interstitial Thermal Therapy (LITT) with hypofractionated radiation therapy for treating newly diagnosed high-grade gliomas.	Recruiting	NCT04699773	[128]

**Table 7:** Patents related to phototherapy for GBM treatment

Patent No.	Claim	Patent date	Applicant
CN113332582B	The claim describes a drug delivery device that integrates an optical element, such as an optical fiber or LED, capable of emitting light directly to the target tissue at the drug administration site. This enables simultaneous phototherapy and drug delivery, allowing light-based treatment (phototherapy) to be precisely applied alongside medication, with real-time monitoring of tissue response through built-in sensors.	07.04.2023	Sun Yat Sen University
CN105983180A	The claims disclose an amphiphilic p-phenoxybenzylamine zinc propionate phthalocyanine compound, its preparation process, and its use as an effective photosensitizer in PDT for treating diseases by light-activated generation of cytotoxic species.	05.10.2016	--
CN201578360U	The claims describe a localized cancer photodynamic therapeutic instrument that utilizes a specially designed LED-based light power irradiator, featuring a Flos Mume dot matrix and a concentric annulus dot matrix of LEDs, controlled by a frequency sweep circuit to adjust emission wavelength, enabling precise delivery of therapeutic light to targeted tissue for effective PDT in cancer treatment.	15.09.2010	--
CN113880849A	The claims describe the synthesis and application of chiral lysine-modified zinc phthalocyanines, which are specifically designed and prepared to serve as photosensitizers in PDT drugs, enabling targeted phototherapy by generating cytotoxic effects upon light activation for the treatment of diseases such as cancer.	04.01.2022	Nanjing Normal University
RU2670087C1	The claims describe the synthesis and formulation of a novel photosensitizer specifically designed for PDT of prostate cancer, where the compound, once administered as part of a drug, accumulates in tumor tissue and, upon targeted light activation, produces cytotoxic effects that selectively destroy cancer cells, thereby utilizing phototherapy as a precise and minimally invasive treatment strategy.	29.01.2018	Mikhail Alexandrovich Green, Nikita Vladimirovich Suvorov, Andrei Fedorovich Mironov
CN108723386A	The claims describe a method for preparing hydrophilic gold nanodendrite particles with strong photothermal conversion efficiency and good biocompatibility, which, when exposed to near-infrared light, generate localized heat capable of killing cancer cells, demonstrating the use of photothermal therapy as a targeted and minimally invasive phototherapy approach in the study.	02.11.2018	Zhejiang University ZJU
JP5873719B2	The claimed conjugates utilize gold nanoparticles functionalized with platinum(II) biradicals to achieve stable, targeted cancer therapy by combining photothermal effects from the nanoparticles with chemotherapeutic action, thereby enabling effective phototherapy.	22.01.2016	--
CN112641946A	The claims describe the preparation and use of polydopamine-coated gold nanocomposites as multifunctional platforms that can load small-molecule antitumor drugs and serve as photothermal agents, enabling combined chemotherapy and photothermal therapy, where the gold nanocomposites, upon near-infrared light irradiation, generate localized heat to kill tumor cells, thus demonstrating the integration of phototherapy for enhanced cancer treatment in the study.	13.04.2021	Suzhou Municipal Hospital
CN107412195B	The claimed method prepares a pH-responsive antitumor drug carrier by coating gold nanospheres (which can serve as photothermal agents for phototherapy) with carboxylated mesoporous silica, loading them with anticancer drugs, and integrating zinc oxide quantum dots, thereby enabling combined phototherapy (via gold nanospheres) and chemotherapy (via drug release) for enhanced cancer treatment.	18.09.2020	Huazhong University of Science and Technology Union Hospital Tongji Medical College Huazhong University of Science and Technology
CN110623940A	The claimed selenium/silica/gold nanocomposite particles are engineered with a mesoporous gold shell that enables efficient photothermal conversion under near-infrared (808 nm) laser irradiation, allowing these particles to be used for photothermal therapy by generating localized heat to kill tumor cells, while also serving as carriers for drug release and as agents for CT imaging.	31.12.2019	Shanghai University of Engineering Science

## Critical analysis and translational approaches

Despite promising preclinical data for PDT, PTT, nanotechnology-based drug delivery systems, and intranasal delivery systems in GBM animal models, several challenges must be overcome before these therapeutic approaches are successfully translated. Thus, in order to systematically evaluate the maturity of these therapies, a technology readiness level (TRL) gap analysis of some of the most promising multimodal phototherapy platforms is presented in Table 7.

**Table 8:** TRL-Gap Analysis of GBM Phototherapy Platforms

Therapy	Nanostructure / Targeting moiety / Dru	Current TRL	Evidence Supporting TRL	Translational Gap	Application	Ref
Pure NIR-PDT	ITIC NPs + DSE-PEG2000 coating	TRL 3 (Proof-of-concept in small animal)	− 739 nm NIR absorbance (deep penetration) - 10.27% ROS quantum yield vs. methylene blue - IC50 7.08 µg/mL + laser; 1.2x tumor growth vs. 10x control - No major organ toxicity (H&E clean)	- Subcutaneous nude mice only (not orthotopic/immune-competent) - No BBB penetration data - Organic semiconductor stability unknown in CSF	Cortical GBM: Surface-accessible tumors post-resection; 739 nm fiber optics through craniotomy	[55]
NIR-II PTT Theranostic	NIR-C12-EL (exosome-liposome hybrid; cyanine dye)	TRL 3 (Subcutaneous efficacy)	1064 nm absorbance; 62.28% PCE; 78.2 °C (9 min); 100% d60 survival; 2.5x tumor accumulation; 0.781% ΦF	Subcutaneous only (no orthotopic/BBB); exosome scalability; 1064 nm clinical fiber integration	Deep GBM (> 1 cm ventricles); NIR-II imaging-guided ablation	[108]
ALA-PDT + EGFR / ABCG2 inhibition	5-ALA (1mM) + lapatinib (0.1µM)	TRL 3 (In vitro human cell line validation)	− 80% viability drop (A172/U118); all lines w/ combo - Pearson correlation-confirmed mitochondrial PpIX shift - Caspase-9/PARP cleavage (H4 cells); ABCG2 efflux blockade	- No animal data; cell-line dependent (H4 > > U87) - Lapatinib poor brain penetration alone - 633 nm shallow penetration limits deep tumors	Fluorescence-guided surgery adjunct: Intraoperative PpIX boost for complete resection margins	[71]
Multimodal PDT / PTT / Ferroptosis + TMZ / ICB	PLGA NPs + ATO + IR780 + GBM exosomes + SPIONs (magnetic targeting)	TRL 4 (Component validated in relevant environment)	- Orthotopic glioma + humanized PDX models: ~50% median survival extension - 2.4x brain accumulation (magnetic-guided) - 82% laser-triggered ATO release vs. 65% passive - 90 μm 3D spheroid penetration (vs. 20 μm free IR780) - 6.27x SOSG (singlet oxygen), 22.95x total ROS - CD8 + infiltration + IFN-γ/TNF-α surge (ICB sensitization)	- No GLP toxicology / pharmacokinetics in larger animals (NHP/canine) - Light delivery dosimetry unscaled for human brain depth (> 3 cm needed) - GMP nano-manufacturing reproducibility - Phase I safety in post-resection GBM patients	Post-surgical residual disease: Intranasal/magnetic-guided delivery for microscopic GBM cells, preventing recurrence via synergistic ICD + ferroptosis + ICB priming	[70]
Chemo-PDT macrophage mimicry	MM@CT (Ce6 + TMZ); macrophage membrane (12.5 nm thick)	TRL 4 (Orthotopic validation)	− 4.9x orthotopic tumor fluorescence; 2.4x half-life - 86% release pH 5.5 vs. 9.4% pH 7.4; CI < 1 synergy - 30% complete regression; 1.4x BBB penetration - 3.4x ROS vs. control; MPS evasion (0.54x liver)	- Macrophage membrane source? (Xenogeneic inflammation risk) - 660–670 nm Ce6 limits depth - TMZ resistance is common in the clinic	Recurrent GBM: Stealth delivery through compromised BBB; TMZ re-sensitization	[72]
Implantable Chemo-PTT	TMZ-PtAu-MOF in chitosan-PCL nanofibers	TRL 3 (Subcutaneous + biocompatibility)	62d TMZ release (pH7.4); 44.5 °C PTT; 0.48 V/V0 tumors; 98% encapsulation; Bax/CASP↑, Bcl-2↓	No orthotopic data; implant logistics; fiber degradation profile	Post-resection cavity filler; 2-month sustained TMZ + PTT	[110]
Intranasal Chemo-PTT	TMZ@GNPs + anti-EphA3	TRL 3 (Subcutaneous + intranasal heating)	IC50 5µM (vs. 1480µM TMZ); 88% tumor inhibition; 40 °C brain ΔT intranasal (vs. 35 °C IV); 281x potency	Subcutaneous only; TMZ-resistant line specificity; no orthotopic survival	TMZ-resistant recurrent GBM: Intranasal rescue therapy	[118]
Intranasal Chemo-PDT	FePt + LND + HA + LF (SPANs)	TRL 3 (Nose-to-brain PK)	Peak brain 60 min post-IN; 26.8pg Fe/cell; >90% release pH5.5; mucus/BBB penetration	No efficacy data (PK only); Fe toxicity concerns; no light activation	Rapid brain loading:	[25]
SDT/PTT/Catalytic Multimodal	BAA-HA (boron nanosheets-AuAgS; HA-CD44 targeting)	TRL 4 (Orthotopic validation)	91% orthotopic regression; 46d median survival; 3.2x BBB (US-microbubble); 90% DPBE↓; 1.8x ¹O₂; catalytic O₂ (0.28mM/min)	US precision for 3 cm tumors; multi-modal dosimetry; boron nanosheet stability	Hypoxic orthotopic GBM; US+laser synergy through intact skull	[109]
Intranasal Chemo-PTT	TMZ16e-GNP-TSL + anti-EphA3 (170 nm, T = 42 °C trigger)	TRL 4 (In vivo survival)	47d median survival (vs. 28d control); 81% release @42°C (vs. 20% @37°C); 6.4 °C brain ΔT; intranasal feasibility; 88% apoptosis	Phase transition precision in heterogeneous GBM; EphA3 expression variability; 780 nm skull penetration	Noninvasive residual GBM: Self-administered intranasal post-resection	[119]
Photobac^®^ Bacteriopurpurin - PDT	IV bacteriochlorin ester	TRL 5 (Phase 1 validation)	NCT05363826 (ongoing Phase I, recruiting, *n* = 24 GBM/gliosarcoma): 8-step dose escalation of IV Photobac^®^ (0.1–2.5 mg/kg) followed by intracavitary NIR-PDT with balloon applicator. Primary endpoints: MTD/DLT; secondary: PK and PpIX fluorescence. Bacteriochlorin enables deeper NIR penetration than porfimer sodium.	Efficacy, light dosimetry optimization	Intracavitary post-resection adjuvant	[125]
Intraoperative 5-ALA PDT (INDYGO trial)	5-ALA + PpIX + intraoperative laser illumination	TRL 6–7 (Phase II feasibility completed with long-term follow-up)	NCT03048240 (INDYGO, completed Phase II pilot, *n* = 30): Maximal fluorescence-guided resection + intraoperative 5-ALA PDT (635 nm laser) + Stupp protocol. Demonstrated excellent safety, feasibility of cavity illumination, adequate PpIX accumulation, and encouraging long-term survival (median OS 23.4 months, 24-month OS 50%, 5-year OS 40%).	Larger randomized controlled trials needed; optimization of light delivery parameters and dosimetry in larger patient cohorts	Intraoperative adjunct to fluorescence-guided surgery in newly diagnosed GBM (post-resection cavity PDT)	[121]
5-ALA PDT	Gliolan^®^ (oral 5-ALA → PpIX)	TRL 9 (Routine clinical use)	Gliolan^®^ (oral 5-ALA → PpIX) holds EMA (2007) and FDA (2017) approval for fluorescence-guided surgery of high-grade gliomas (routine clinical use). INDYGO trial (NCT03048240) confirmed safety and feasibility of intraoperative PDT post-resection. Ongoing NCT05850377 evaluates long-term DFS.	Efficacy beyond surgical guidance (systemic/recurrent)	Intraoperative fluorescence-guided resection + per-resection PDT	[59]

From the data in Table 7, it can be concluded that most innovative nanotechnology-based drug delivery platforms, such as magnetic-targeted multimodal PDT/PTT/ferroptosis, macrophage membrane-camouflaged chemo-PDT, and intranasal chemo-photothermal therapy, have shown significant efficacy in orthotopic glioma models and humanized patient-derived xenograft (PDX) models. The efficacy has been indicated by a survival advantage of ~ 50%, 2.4-fold accumulation of these platforms in the brain following magnetic guidance, increased drug release using a laser, penetration of these platforms into tumors, significant levels of ROS generation, and infiltration of CD8 + T cells, along with cytokine surges. Thus, these platforms have shown significant promise for combining phototherapy with TMZ, ICB sensitization, and ferroptosis induction for the treatment of residual tumor tissue following resection and for the prevention of recurrence. On the other hand, more clinically advanced PDT platforms, such as 5-ALA Gliolan, have demonstrated significant efficacy in achieving a TRL of 9 in fluorescence-guided surgery. PDT has demonstrated significant efficacy in achieving a TRL of 6–7 in intraoperative PDT in the context of the INDYGO Phase II clinical trial, which has demonstrated safety, efficacy, and sufficient PpIX accumulation. However, PDT platforms have not demonstrated significant survival benefits in GBM patients, which is largely due to the difficulty in light dosimetry in the GBM microenvironment. Similarly, porfimer sodium-based PDT has demonstrated significant efficacy in achieving a TRL of 7 in Phase III clinical trials. From these comparisons, it is evident that both modalities have their advantages and disadvantages. PDT, for instance, requires oxygen for the generation of ROS, which is lacking in GBM due to its hypoxic nature. Moreover, light penetration is limited to 0.5 cm. PTT, on the other hand, allows for tumor ablation in the absence of oxygen through hyperthermia. Moreover, NIR has the advantage of deeper tissue penetration, up to 2–3 cm. However, there is the risk of thermal damage to adjacent neural tissue. Thus, temperature control is essential in this modality. Moreover, tumor selectivity is very high. Regarding the use of nanotechnology in drug delivery, there has been a significant improvement in the penetration of these agents into the tumor. In fact, tumor penetration has been shown to be 5-fold or more than that of free agents. Moreover, there has been a significant improvement in the ability of these agents to cross the BBB. However, there are issues related to nanoparticle toxicity, biodistribution, immunological clearance, and reproducibility of GMP-scale production. Thus, most of these platforms are in TRL 3–5, far from the maturity level of TMZ, which is TRL 9. The use of intranasal administration has been shown to provide a non-invasive means of drug delivery to the CNS. It has been shown to bypass the BBB. Moreover, there is an improvement in the brain exposure of drugs. Furthermore, there is improvement in the survival of animals. However, the efficiency of this mode of administration is limited by mucociliary clearance.

The combination of the specific mechanisms of action of PDT and PTT, which include ROS-dependent cell death and immunological priming, and oxygen-independent and deeper tissue penetration of PTT, respectively, has prompted the design of multimodal systems that combine phototherapy with chemotherapy, immunotherapy, and ferroptosis induction [129]. In our opinion, TRL-4 level platforms that include intranasal and magnetic guidance systems, catalytic oxygen production, and multimodal cell death mechanisms have the greatest potential for development and use as adjuvant therapy. Such approaches may have significant potential for treating microscopic residual GBM cells and overcoming the drawbacks of individual modalities. However, for these approaches to be brought to the clinic and demonstrate their potential as adjuvant therapies for patients suffering from GBM, GLP toxicology and PK studies in large animal models (non-human primates and/or dogs), standardized multi-modal light dosimetry protocols for depths in the human brain greater than 3 cm, reproducible GMP production of these approaches, and thoughtfully designed adaptive Phase I/II clinical trials with real-time imaging guidance will be necessary. Collaboration across nanotechnology, neurosurgery, and photobiology will be necessary to bridge the gap between these approaches and the current standard of care, the Stupp protocol.

## Future directions and challenges

Phototherapy, which includes PDT and PTT, is becoming a promising modality for the treatment of GBM, a very aggressive brain tumor, with few effective treatment options. Current studies are developing drug delivery platforms, nanotechnologies and photoactivation modalities, hence enhancing the specificity and effectiveness of phototherapeutic treatments and creating more specific, less toxic treatment regimens. The use of nano-implementors includes emerging technologies, such as smart nanocarriers and implantable light-emitting devices, with the aim of improving targeted delivery and reducing collateral cytotoxicity in normal brain tissue. One promising research direction includes intranasal delivery, which takes advantage of the olfactory and trigeminal routes and circumvents the BBB by offering an effective noninvasive target of PSs and nanoparticles to the CNS. Nevertheless, there are still considerable obstacles that affect clinical performance, such as fast mucociliary clearance, poor retention of drugs, and nonhomogeneous tumor distribution. In addition, aligning PS delivery with photonic activation is also an issue through the intranasal route, which reduces the overall accuracy of PDT.

Although phototherapy has reduced systemic toxicity because it is minimally invasive and has a localized effect, there are significant challenges that need to be overcome to realize successful clinical translation. The shallow depth at which visible and NIR light can penetrate brain tissue, often requiring interstitial or implantable light sources, is a major weakness and causes further complications during the procedure. Moreover, the accumulation and retention of therapeutic agents in GBM tissue are still not efficient because tumor heterogeneity and the tumor microenvironment are difficult to address. Patient-specific variables, such as tumor location, genetic makeup, immune status, and oxygenation, further complicate the standardization of treatment. These factors can influence drug uptake, light absorption, and overall response to therapy, underscoring the need for personalized treatment plans. The personalization of treatment regimens with respect to tumor characteristics, including the choice of PS, light dose and light delivery route, is crucial for achieving optimal results. The combination of phototherapy with adjunctive therapies such as chemotherapy and immunotherapy can also have synergistic effects. For example, PDT can promote immunogenic cell death, supporting immune-based treatments, whereas PTT can increase tumor permeability to aid drug uptake. Recent initiatives in nanotechnology are indicated in patent applications, which show that the evolving interest in this area is shared by scholars and industry and that current applications are focused on multifunctional nanoparticles, intranasal delivery platforms, and advanced photo-optical devices. These inventions are indications of a strong driving force to overcome the existing obstacles in the effective management of GBM through phototherapy.

## Conclusion

Phototherapy remains a prospective intervention approach for GBM, which is not only innovative in its underlying mechanistic principles but also opens novel opportunities in the adoption of modern therapeutic approaches in combination with precision medicine and nanotechnology. Currently, the main principles and initial processes are firmly established, but the route to consistent clinical implementation necessitates a tactical turnaround in which the conceptual endorsement is substituted by integrative application in the context of current therapeutic strategies. To maximize the potential of phototherapy, the remaining bottlenecks, such as optimization of the clinical protocol and improvement of delivery consistency, as well as aligning treatment timing to the tumor profile in an individual, need to be resolved in the current research. Instead of focusing on phototherapy as an independent treatment, its future efficacy might be based on its integration into multimodal therapy comprising surgery, chemotherapy, immunotherapy, and diagnostics.

It is also necessary to promote interdisciplinary work in scientific, medical, and regulatory discussions. Such a concerted effort will hasten the process of converting theoretical knowledge gained through experimentation to actual practice so that breakthrough technologies are not slowed down owing to translational barriers. In this venture, strategic alliances, transparent regulatory guidelines, and strong clinical trial frameworks will be of key importance. The future of phototherapy in the clinical management of GBM is perhaps dependent not only on its single-agent efficacy but also on its ability to improve and support larger therapeutic interventions. With continued refinement and interdisciplinary momentum, the therapeutic landscape of this formidable disease could be significantly reshaped.

## Data Availability

No datasets were generated or analysed during the current study.
